# The ecology of gestational growth in a wild cooperative mammal

**DOI:** 10.1111/1365-2656.70199

**Published:** 2025-12-12

**Authors:** Jack Thorley, Tim Clutton‐Brock, Helen C. Spence‐Jones, Zoe Turner, Stuart P. Sharp, Marta B. Manser, Winnie Boner, Robert Gillespie, Dominic L. Cram

**Affiliations:** ^1^ Department of Zoology University of Cambridge Cambridge UK; ^2^ Kalahari Research Centre Kuruman River Reserve Van Zylsrus South Africa; ^3^ Mammal Research Institute University of Pretoria Pretoria South Africa; ^4^ Helmholtz Centre for Polar and Marine Research Alfred‐Wegener‐Institut List auf Sylt Germany; ^5^ Department of Evolutionary Biology and Environmental Studies University of Zurich Zurich Switzerland; ^6^ Lancaster Environment Centre Lancaster University Lancashire UK; ^7^ College of Medical, Veterinary and Life Sciences, Institute of Biodiversity, Animal Health and Comparative Medicine University of Glasgow Glasgow UK; ^8^ Centre for Ecology, Evolution and Conservation, School of Biological Sciences University of East Anglia Norwich UK

**Keywords:** gestation, gestational weight gain, maternal investment, meerkat, pregnancy, prenatal growth, telomeres

## Abstract

1. In wild mammals, early postnatal growth strongly affects offspring survival and fitness, but little is known about the causes and consequences of variation in *prenatal* growth.
2. We investigated whether gestational weight gains vary according to maternal traits and social and environmental conditions, and how prenatal growth affects the fates of the resulting offspring, using an exceptionally large sample of repeated pregnant body weight records from individually recognizable wild meerkats (*Suricata suricatta*).
3. Pregnant meerkats' body weights remained stable during the first half of gestation and then increased linearly until they gave birth.
4. Gestational weight gains were more rapid under favourable environmental conditions and when mothers were experimentally food‐supplemented, suggesting that nutrition strongly determines prenatal growth.
5. While social conditions and reproductive competition shape *postnatal* growth in many social vertebrates (including meerkats), these factors had a limited effect on prenatal growth, and adjustment to gestation lengths were modest and unrelated to social factors.
6. Pups that grew faster in utero were heavier when they emerged from the birth burrow yet this rapid growth was not associated with shortened leukocyte telomeres, and they were consequently more likely to survive to adulthood.
7. Broadly, we identified pronounced variation in gestational weight gains, which is largely driven by food availability and strongly predicts offspring birth weights and survival.
8. Our findings also highlight constraints in the flexibility of prenatal growth and gestation lengths in this species, which may limit adjustments in response to prevailing social conditions, and enhance selection for flexibility in postnatal growth.

## Graphical abstract

Using data from hundreds of wild pregnant meerkats, Thorley et al show that gestational growth varies widely, is shaped by nutrition but not social conditions, and improves pup survival without shortening telomeres. The fitness consequences and limited flexibility of prenatal growth likely enhance selection on early postnatal growth plasticity.

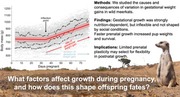

## 1 INTRODUCTION

In wild mammals, birth timing and birth weight can have important consequences for offspring development, reproductive success and survival, and both depend on the rate and duration of foetal growth (Guinness et al., 1978; Maniscalco, 2014; Post, 2003). While the causes and consequences of variation in *postnatal* growth have been investigated in several long‐term studies of wild mammals (Allainé et al., 1998; Altmann & Alberts, 2005), less is known of the factors that shape foetal growth and gestation lengths and their consequences for resulting offspring (Clements et al., 2011; Inzani et al., 2016; Viblanc et al., 2016).

Foetal growth can be challenging to directly measure in wild mammals, but sequential maternal body mass records during pregnancy can offer a reliable proxy (Sharp et al., 2013). In medical literature, this is referred to as ‘gestational weight gain’ (GWG), and though it comprises both foetal and other tissues (e.g. maternal fat reserves and mammary tissues for lactation), GWG tightly predicts birth weights in humans, non‐human primates, and livestock (Baumgaertner et al., 2024; Hutcheon et al., 2019; Price et al., 1999; Wallace et al., 2021). Moreover, while precise measurement of gestation length in wild mammals is challenging due to difficulties in observing conception and birth dates, the duration of the period in which most foetal growth occurs can be estimated by identifying the onset of GWG in mothers. Field studies providing repeated measures of maternal body mass during gestation consequently provide a rare opportunity to estimate foetal growth and gestation lengths under natural conditions.

Gestation lengths and GWGs are likely to be affected by environmental factors which affect food availability. In humans and livestock, GWGs increase with improvements to maternal nutrition (Kramer & Kakuma, 2003; Mellor, 1983). Gestation lengths may also vary according to prevailing environmental conditions, as females time their births and subsequent lactation in relation to food availability (determined by temperature, rainfall and local primary productivity), predation risk or to re‐conceive quickly (Estes, 1976; Friebe et al., 2014). As such, environmental factors can have striking effects on the rate and duration of GWGs in humans and livestock, and in wild mammals these effects are likely to be stronger because of unpredictable food availability.

Gestation lengths and GWGs may also change in response to the social environment. In many social vertebrates, postnatal growth can be adjusted to enhance competitiveness or minimize aggression from group‐mates (Buston & Clutton‐Brock, 2022), but it is not yet known whether foetal growth is similarly responsive to the social environment. Prenatal growth could be accelerated in response to predicted levels of competition offspring will face (Inzani et al., 2016), or reduced if mothers anticipate enhanced alloparental care (Dixit et al., 2017; Sharp et al., 2013). In some species, gestation lengths appear to vary in relation to reproductive competition. Where pregnant females kill young that would compete with their own (Lukas & Huchard, 2019), mothers synchronize births so that infanticidal females would risk killing their own offspring (Hodge et al., 2011). As such, the social environment may impose strong selection for plasticity in foetal investment and gestation lengths.

Variation in GWG and gestation lengths are likely to have lifelong consequences for offspring, though these effects remain largely unexplored in wild mammals. Sub‐optimal birth timing and low birth weights arising from weak GWGs can generate long‐lasting harmful health effects (Barker, 1998; Plard et al., 2015). In field studies, the long‐lasting consequences of early adversity are often investigated through their effects on proxy measures such as telomere lengths, which estimate biological state without the need to follow individuals for their entire lives (Ridout et al., 2017). Telomeres are sequences at the ends of eukaryotic chromosomes that shorten with each cell division, and shortening is associated with reduced survival in many species (Cram et al., 2017, 2018; Wilbourn et al., 2018). Telomere loss is accelerated during postnatal growth (McLennan et al., 2016; Ringsby et al., 2015), but whether prenatal growth similarly shortens telomeres is unclear.

Here, we investigate whether GWGs and gestation lengths vary with environmental and social conditions in wild Kalahari meerkats (*Suricata suricatta*), using repeated body mass records from individually recognisable pregnant females. We also examine how GWGs predict postnatal development and survival of the resulting young. Meerkats are well suited to addressing these research questions, for four reasons. First, environmental conditions, such as air temperature and local primary productivity (measured as normalized difference vegetation index [NDVI]) in the Kalahari Desert strongly influence postnatal growth and reproduction, suggesting similar effects on prenatal growth (Groenewoud & Clutton‐Brock, 2021; Van de Ven et al., 2020). Second, as obligate cooperative breeders, social factors strongly influence reproductive success (Russell et al., 2003). Within each group, reproduction is largely monopolized by the dominant female, but subordinates breed at a lower frequency (Griffin et al., 2003). There is consequently intense reproductive competition, which manifests as infanticide of rivals' newborn litters by pregnant females, and eviction of subordinates by the dominant female (Clutton‐Brock et al., 2010). Flexibility in GWGs and gestation lengths could minimize the costs of this reproductive competition. Third, meerkats show pronounced plasticity in *postnatal* growth in response to social conditions (Huchard et al., 2016). As such, there are adaptations to adjust postnatal growth according to social conditions, and selection pressures to do so for prenatal growth, but whether this occurs is unclear. Fourth, early‐life postnatal growth has long‐lasting consequences for survival and fitness (English et al., 2013), suggesting that in utero growth may be similarly consequential.

Specifically, we first characterize the onset, duration, and rate of GWG in high resolution for hundreds of pregnancies under natural conditions. Second, we identify the environmental, social, and maternal determinants of these aspects of GWG, and experimentally test the effects of maternal nutrition. Third, we use a subset of pregnancies with known conception dates to investigate whether gestation lengths are extended during periods of intense reproductive competition to avoid infanticide, or curtailed during favourable environmental conditions to allow females to quickly re‐conceive. Finally, we investigate how the duration and rate of GWG affect the postnatal body mass and telomere lengths of the resulting offspring, and their survival to nutritional independence and 1 year.

## 2 METHODS

### 2.1 Meerkat life‐history and body mass data

We studied wild meerkats in the context of a long‐term, individual‐based study at the Kuruman River Reserve in South Africa (26°58′ S, 21°49′ E) (Clutton‐Brock & Manser, 2016). Meerkats live in groups of 15 ± 7 individuals (mean ± 1 standard deviation [SD]; Duncan et al., 2023). Most individuals were of known age and could be weighed on electronic scales before they began foraging in the morning, in return for water or a crumb of boiled egg. Individuals were identified using unique dye marks and subcutaneous transponders (Identipet, Johannesburg, South Africa), and each group's dominant female could be identified because she received submissive behaviours from other females. Groups were visited three to four times per week to record group composition and life‐history events.

Pregnancies were identified by swelling of the abdomen, and were deemed successful by the presence of babysitters at the birth burrow following a large drop in maternal body mass. The date of birth was assigned as the night before a female was observed no longer pregnant (with a mean uncertainty of 0.84 ± 1.02 days). We estimated that meerkat pregnancies are 75 days in length based on published estimates and inter‐birth intervals in our population (Spence‐Jones et al., 2021; Supporting Information S1) and back‐dated conception dates from birth. GWG is likely to tightly reflect foetal growth in this species, because almost all of the mass gained during pregnancy is lost after birth (and as such, little is retained as fat reserves for lactation or other purposes, Supporting Information S2).

We maximized the resolution with which we could characterize body mass changes by including only pregnancies with at least four measurements in each 25‐day trimester, as some pregnancies below this threshold had long periods without data. This resulted in 381 pregnancies from 135 females in 46 groups from 1998 to 2022, most of which far exceeded this threshold: on average, females were weighed 31.8 ± 8.9 times per pregnancy, or approximately every 56.6 h (*n* = 12,104 body mass records). Dominant females comprised 81% of the pregnancies (*n* = 307 pregnancies from 84 dominants; *n* = 74 pregnancies from 63 subordinates). Sixty‐six females were present in the dataset for more than one pregnancy (range 1–21 pregnancies per female, 2.82 ± 3.10 [mean ± 1 SD]).

Litter size strongly influences GWG in meerkats (Sharp et al., 2013), so we only included pregnancies in which the pups could be counted after emerging from the natal burrow (ca. 2–3 weeks old). Our results therefore reflect pregnancies producing surviving young and may not apply to those ending in abortion. The average litter size of dominant and subordinate females was 3.65 ± 1.38 and 3.20 ± 1.16 (mean ± 1SD), respectively. We used an ultrasound scanner (Sonoscape S6, Sonologic, Shenzhen, China) to confirm that where litters are not entirely lost (likely due to infanticide, Cram et al., 2019), emerging litter size accurately reflects in utero litter size (68 pups emerged from 69 detected embryos in 29 pregnancies, average pre‐emergence loss per litter: 0.06 ± 0.56 pups, Supplementary Materials S3).

### 2.2 Modelling gestational weight gains

**FIGURE 1:**
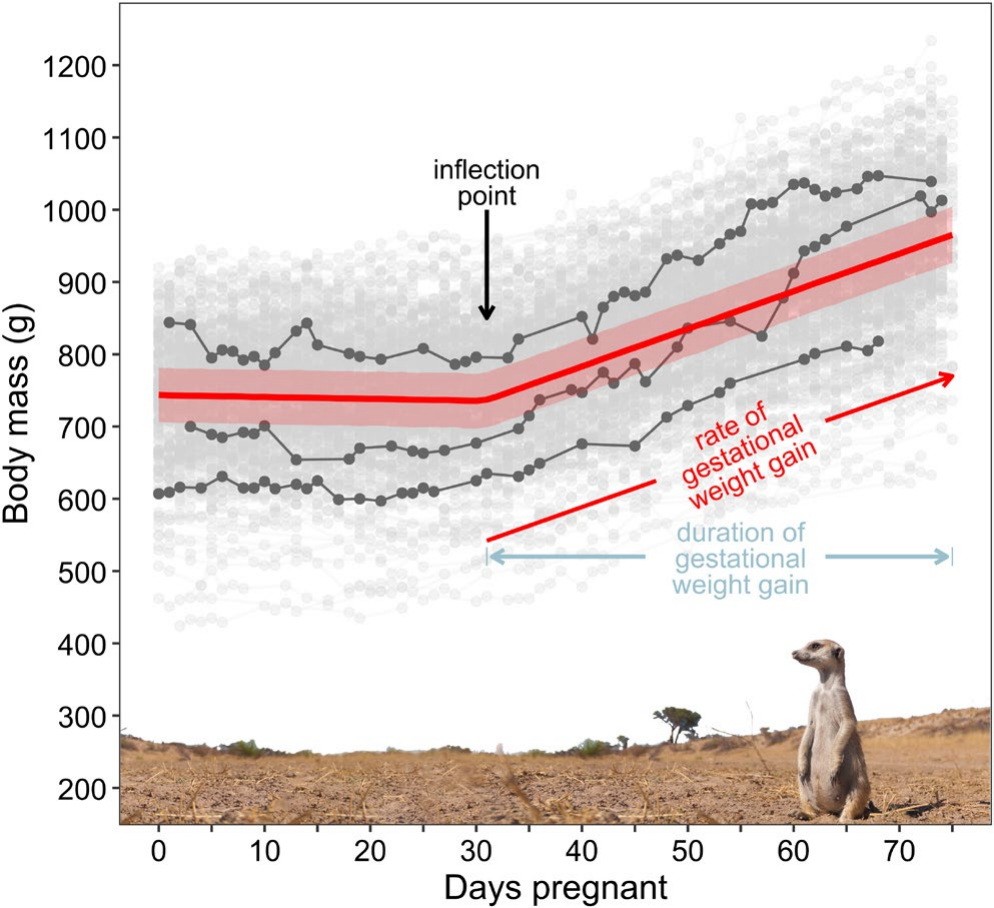
Changes in body mass in pregnant wild meerkats. The body mass data for all pregnancies is plotted in grey (*n* = 381), with three representative pregnancies highlighted in dark grey to show among‐pregnancy variation. The predicted population‐level mean body mass (Model 1) is plotted in red, with shaded bands indicating the uncertainty in predictions when accounting for population‐level ‘fixed’ effects and the observation‐level ‘residual’ variance (red band; ±95% credible intervals [CrI]).

Prior work has shown that meerkat GWG is biphasic: body mass is stable for the first half of pregnancy, and increases linearly thereafter until parturition (Sharp et al., 2013). To capture this biphasic shape, we modelled GWGs using piecewise linear models with an inflection point (Figure 1), implemented within a mixed effects framework. This approach allowed us to estimate population‐level changes in the growth trajectory, controlling for pregnancy‐specific variation in the onset and rate of GWG through random effects. We outline the models below (full details including priors are in Supporting Information S4).

#### 2.2.1 How does maternal body mass change through gestation?

In Model 1, we characterized the shape of GWG by resolving the intercept (body mass at conception), inflection point, and pre‐ and post‐inflection slopes at the pregnancy and population levels. We included pregnancy‐level random effects and litter size as a fixed effect on both the inflection point and the post‐inflection slope, because of the prior expectation that litter size would influence the rate and onset of GWG. We also estimated the correlations among the pregnancy‐level random effects.

In Model 2, we included random effects of female identity and breeding season for each shape parameter, to examine whether pregnancy‐level correlations among the shape parameters changed after controlling for these random terms. We calculated the duration of GWGs by subtracting the estimated inflection point from 75, and translated covariate effects on inflection timing (see below) into effects on GWG duration by multiplying their estimates by −1.

#### 2.2.2 What factors are correlated with gestational weight gain dynamics?

In Model 3, we extended Model 2 to examine whether maternal, social, and environmental factors were associated with variation in GWG. Specifically, we examined whether the covariates of interest affected the body mass at the start of gestation, the inflection point and the rate of GWG (the post‐inflection slope). For each of these three shape parameters we included as predictors: litter size, dominance status (dominant or subordinate), the mother's age at conception and its quadratic (to control for contrasting body mass changes in younger and senescent females), group size (the average number of adults in the group in the 30 days prior to conception) and its quadratic, and the number of other females pregnant in the group at the same time (the number of pregnant females in their third trimester during the focal female's third trimester). For the inflection point and the post‐inflection slope we also included covariates for maternal body condition at conception (Supporting Information S5), primary productivity of the environment (NDVI), and air temperature (Supporting Information S6) to capture variation in the quality of mothers and environmental conditions, respectively. To test whether dominant and subordinate mothers differed in their *per capita* investment in developing young, and in their responses to reproductive competition, we included interactions between dominance status and litter size and between dominance status and the number of other pregnant females.

All models were fit in brms 2.17.0 (Bürkner, 2018), an R interface for fitting Bayesian statistical models in Stan (Carpenter et al., 2017), using R version 4.2.2 (R Core Team, 2024). All continuous predictors were centred and scaled to 1SD prior to model‐fitting. The datasets and code necessary to reproduce our analyses are in the Supporting Information, along with a summary of all the piecewise models fit to body mass data (Table S2). To interpret the strength and the uncertainty of effects we inspected the posterior means and 95% credible intervals (CrI). To assess the biological importance of effects we calculated the *probability of direction*, which indicates the proportion of the posterior distribution that matches the median's sign (*p*_+_/*p*_−_) (Makowski et al., 2019). We interpret parameters with *p*_+_/*p*_−_ < 0.90 as being highly unlikely to exist, 0.90 < *p*_+_/*p*_−_ < 0.95 as having a moderate probability of existing, and *p* > 0.95 as having a high probability of existing.

#### 2.2.3 How does supplementary feeding affect the duration and rate of gestational weight gain?

We used data from a supplementary feeding experiment to investigate the role of maternal nutrition in GWGs (Dubuc et al., 2017). Dominant females were randomly assigned ‘fed’ or ‘unfed’ (control) treatments. ‘Fed’ females were given one hard‐boiled hen's egg per day commencing 6 weeks after the end of one pregnancy and continuing until they gave birth to the focal pregnancy. Thereafter, they received four eggs per week until weaning. Control females were approached to a similar degree but not fed. The final dataset included nine pregnancies in each treatment (Supporting Information S7). To model the effect of food supplementation, we fit a version of the GWG model including effects of treatment (fed/unfed) and litter size on both the inflection point and the post‐inflection slope (Model 4). A random effect of pregnancy was included for all shape parameters.

#### 2.2.4 Do mothers adjust their gestational lengths?

For a subset of pregnancies, gestation length could be calculated accurately as the period between observed oestrus or copulation, and subsequent birth. During oestrus, dominant males followed females closely, sniffing the anogenital region and attempting to copulate. Pregnancy lengths were available for 76 pregnancies from 40 females (*n* = 2526 body mass measures). To determine whether gestation lengths were responsive to environmental and social conditions, we fit a standard linear model with gestation length as the Gaussian response and the following predictors: litter size, body condition at conception, group size, the number of other females pregnant concurrently, NDVI, and temperature, which we included as both a linear and quadratic term. Female identity was included as a random effect. Six pregnancies commenced before NDVI data were available; this model was therefore fit to 70 pregnancies.

We also fit a piecewise model to this dataset (Model 5) to determine how longer pregnancies were achieved. Pregnancy extensions could feasibly occur by increasing the period from conception to the onset of GWG, by increasing the duration of GWG, or both. For the 76 pregnancies, we reworked the time variable so that the day of pregnancy reflected the time from oestrus/copulation (Day 0). We then fit a version of the piecewise model that included effects for gestation length and litter size on both the inflection point and the post‐inflection slope. We also included random effects of pregnancy identity (a unique identifier for each pregnancy) and female identity for all the shape parameters. We did not standardize the gestation length term in this model to facilitate interpretation of results. Consequently, the parameter estimate reflects the relative position of the inflection point for a 1‐day increase in gestation length: values >0.5 imply that increases in gestation length are associated with a proportionally greater lengthening of the pre‐inflection period relative to the post‐inflection period, while values <0.5 imply the reverse.

We measured inter‐pregnancy intervals (IPI, the time between the end of one pregnancy and the conception of the next) following known‐length gestations. For full‐term second pregnancies, we estimated the conception date as 75 days before birth. For second pregnancies that ended in abortion, we conservatively estimated the conception date as 35 days before pregnancy loss (as pregnancies are typically detected 40 days before birth in our population, equating to 35 days after conception). Of the 76 known‐length pregnancies, 70 were followed by another pregnancy (IPI: 34.5 ± 53.1 days, range = 1–221 days; the remaining six were those mothers' final pregnancies). To examine whether shorter gestation lengths were associated with rapid subsequent conceptions, we fit a linear model with gestation length as a predictor of IPI.

#### 2.2.5 How do gestational weight gains affect pup fates?

To explore the fitness consequences of variation in GWG, we fit a simplified version of Model 1 that excluded litter size effects and extracted estimates for the duration and rate of GWG for each pregnancy. We converted the estimates of the rate of GWG to *per capita* growth rates by dividing each post‐inflection slope by the litter size. These estimates were then included as predictors in secondary linear mixed effects models that tested whether the duration and/or rate of GWG predicted pups' (i) body mass (Gaussian response) and (ii) leukocyte relative telomere lengths at emergence from the birth burrow (Gaussian response, Supporting Information S8), (iii) survival to nutritional independence at 90 days and (iv) survival to adulthood at 1 year (both Bernoulli response). These models included data from 971 pups (289 litters), 159 pups (42 litters), 960 pups (287 litters) and 945 pups (283 litters) respectively, according to data availability.

Each model included the following population‐level effects: the estimated duration of GWG, the estimated *per capita* prenatal growth rate, the mother's age (in days, and its quadratic), the mother's dominance status and group size at birth, and the following random effects: pregnancy identity, female identity and breeding season. For the pup emergence body mass model, we also included the age at which this measure was taken. For the relative telomere length model, we included the age at which the skin tissue sample was taken, and excluded the breeding season random effect as individuals were sampled in only two seasons. For models of pup survival, we also included pup emergence body mass, which strongly influences early‐life survival in meerkats (Cram et al., 2017). As these data were from pups ranging in age (12–30 days old), we calculated each pup's expected body mass at 25 days (Supporting Information S9). We accounted for uncertainty in parameter estimation for the growth duration and post‐inflection slope for each pregnancy by drawing 100 random samples from the posterior distribution, modelling these 100 datasets individually and combining the posterior estimates (Supporting Information S10).

## 3 RESULTS

### 3.1 How does maternal body mass change through gestation?

The average female weighed 739.70 ± 86.09 g at conception, and 943.52 ± 103.05 g on the final day of pregnancy (means ± 1 SD). Our models indicated that a typical pregnant female lost 0.26 g/day from conception until GWG commenced (Model 1, *β* = −0.26 [SD = 0.11], 95% CrI: [−0.48, −0.04], *p*_−_ = 0.99; Table S3, Figure 1). After this inflection point, she gained 5.20 ± 0.08 g/day (Model 1, 95% CrI: [5.04, 5.37], *p*_+_ = 1.00), and this rate increased by 0.66 ± 0.09 g/day for every additional foetus (Model 1, 95% CrI: [0.49, 0.83], *p*_+_ = 1.00, litter size mean = 3.57 ± 1.35 pups). The mean onset of GWG was 31 days after estimated conception (Model 1, *β* = 30.83 [SD = 0.49], 95% CrI: [29.87, 31.79]); the mean duration of GWG was therefore 44 days, which our model was able to resolve with 95% credible intervals spanning less than 48 hours (95% CrI: [43.21, 45.13]). Our models also detected correlations among pregnancy shape parameters and maternal body mass at conception (Supporting Information S11, Tables S1, S3 and S4 and Figure S2).

### 3.2 What factors are correlated with body mass changes during pregnancy?

**FIGURE 2:**
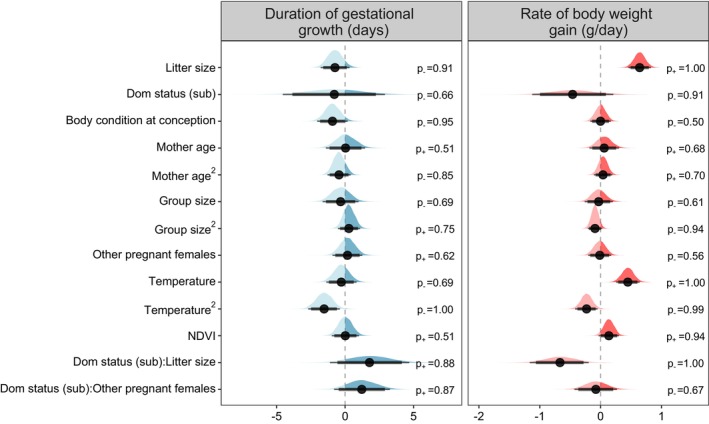
The effect of maternal, social, and environmental factors on gestational weight gain parameters in wild meerkats. Factors were modelled against three components of gestational weight gains: the average body mass at the start of gestation (Supporting Information S12), the timing of the onset weight gain (presented as the duration of gestational weight gain), and the rate of gestational weight gain. The full posterior distribution for each model estimate is presented, with point intervals providing the mean, 89% and 95% credible intervals. Squared predictors refer to those fit as quadratic terms, and parentheses refer to factor reference levels. The probability of direction (positive: *p*_+_; negative: *p*_−_) is indicated to the right of each distribution.

**FIGURE 3:**
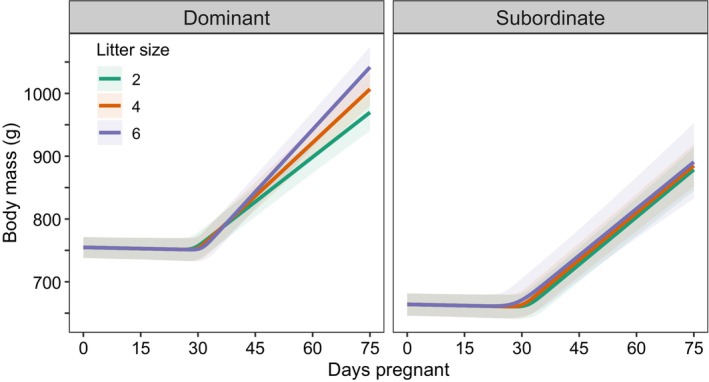
The effect of litter size on gestational weight gains in dominant and subordinate meerkats. Dominant females accelerated their gestational weight gain when gestating larger litters, while subordinate females did not (dominants: *N* = 309 pregnancies from 85 females; subordinates: *N* = 74 pregnancies from 63 females). Lines display the predicted body mass of mothers with litter sizes of 2, 4 and 6, with shaded bands indicating the uncertainty after accounting for population‐level ‘fixed’ effects only (±95% credible intervals). All other continuous predictors were held at their status‐specific population means.

**FIGURE 4:**
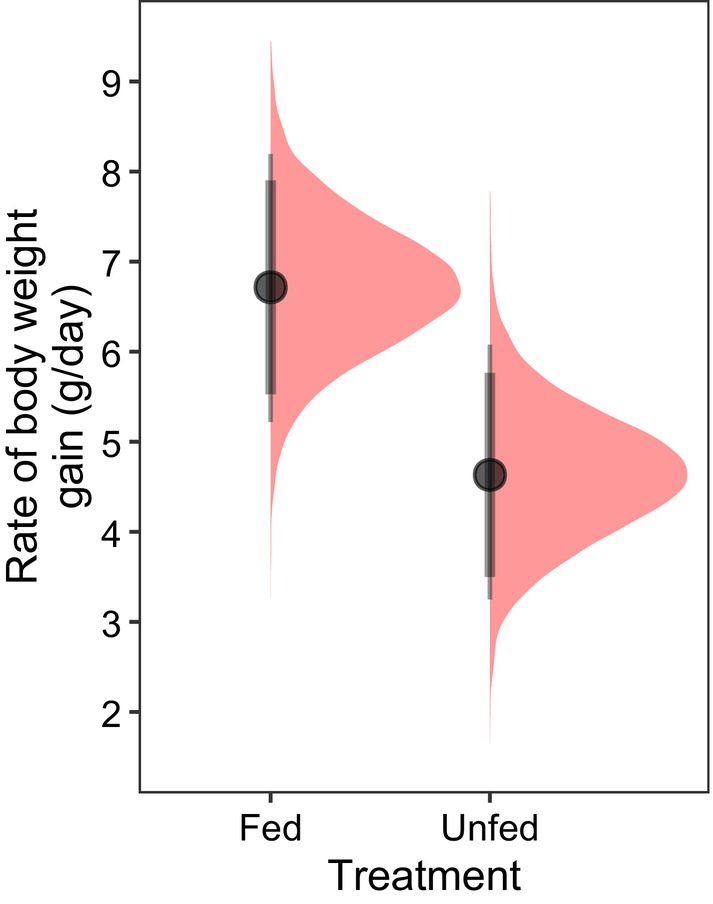
The effect of food supplementation on the rate of gestational weight gain in wild meerkats. Pregnant females given additional food gained more body weight than control (unfed) females. The full posterior distributions are shown in red for each treatment (*n* = 14 females) with point intervals providing the mean, 89% (thick error bars) and 95% (thin error bars) credible intervals. The posteriors incorporate the population‐level ‘fixed’ effects only and reflect the predicted gestational weight gain at the mean litter size of 3.24.

The rate of GWG was faster as temperatures increased, before levelling off at 32°C (quadratic effect of temperature, Figure S9). GWGs also accelerated following periods of high local primary vegetation productivity (*β* = 0.14 [SD = 0.09], 95% CrI: [−0.03, −0.30], *p*_+_ = 0.94). Model 3 recovered the positive effect of litter size identified in Models 1 and 2 but highlighted that this was restricted to dominant females (*β* = 0.64 [SD = 0.09], 95% CrI: [0.46, 0.83], *p*_+_ = 1.00). Subordinate females, by contrast, did not gain mass faster when gestating larger litters (*β* = −0.03 [SD = 0.23], 95% CrI: [−0.49, 0.41], *p*_−_ = 0.54, Figure 3). The rate of GWG was unrelated to the maternal body condition at conception, group size or its quadratic term, or the number of females concurrently pregnant (Figure 2, Table S5). In the supplementary feeding experiment, mothers receiving additional food gained body mass faster than control (unfed) mothers (Model 4, *β* = 2.12 [SD = 1.03], 95% CrI: [0.04, 4.12], *p*_+_ = 0.98, Figure 4, Table S6). We found no evidence that GWGs were extended or shortened by supplemental feeding (*β* = 1.27 [SD = 3.15], 95% CrI: [−4.93, 7.49], *p*_+_ = 0.66).

The duration of GWG was negatively associated with body condition at conception (*β* = −0.92 [SD = 0.57], 95% CrI: [−2.04, −0.19], *p*_−_ = 0.95), such that a 50 g increase in conception body condition (ca. 7% increase in total body mass for an average mother) was predicted to reduce the duration of growth by 1.16 days (95% CrI: [−2.58, −0.23], Supporting Information S13). The period of GWG was longest at intermediate temperatures (ca. 29°C) and declined in cool or extremely hot conditions (Figure S9). In subordinates, there was a small, weakly supported effect of GWG being extended when multiple females were breeding concurrently (*β* = 1.39 [SD = 0.91], 95% CrI: [−0.34, 3.16], *p*_+_ = 0.94), while no such effect was seen in dominants (Figure 3, Table S5).

**FIGURE 5:**
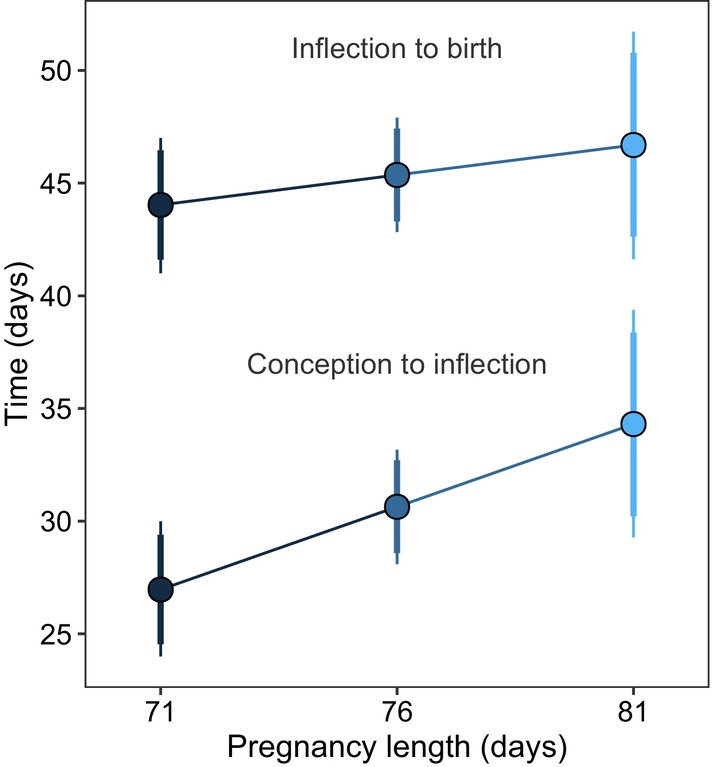
Constraints on meerkat pregnancy lengths before and after the onset of gestational weight gains. For known‐length pregnancies (in which conception and birth were observed, *n* = 76 pregnancies from 40 females), longer pregnancies were achieved primarily by mothers extending the period from conception to the onset of gestational weight gains (‘inflection’), rather than by delaying giving birth (i.e. extension of the period from inflection to birth). Lines represent the predicted duration of the two phases of gestation (±89% (thick) and 95% (thin) credible intervals).

**FIGURE 6:**
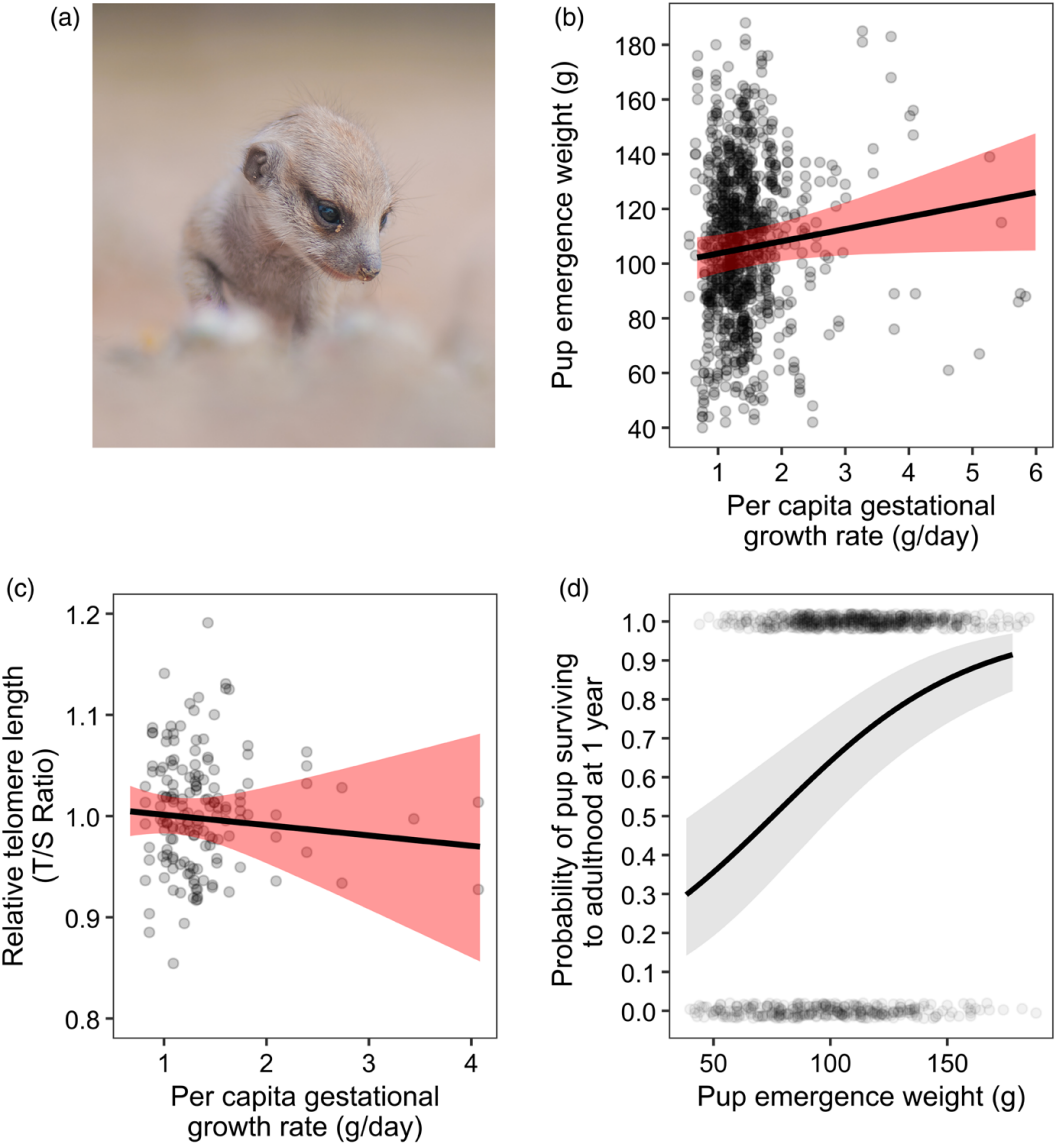
The fitness consequences of variation in prenatal growth on offspring development. (a) A meerkat pup emerging from the burrow at 3 weeks old. (b) Increases in pups' prenatal growth rates (estimated as the *per capita* gestational growth rate) were associated with increased body mass at emergence from the birth burrow, but (c) did not lead to pups having shorter leukocyte relative telomere lengths. (d) Pups that emerged heavier displayed higher survival to adulthood. Solid lines display the predicted mean response for dominant females when all other model covariates were held at their mean, with shading highlighting the 95% credible intervals conditional on population‐level ‘fixed’ effects. Points display the raw data (d) or the mean posterior estimates (b, c).

### 3.3 Can mothers adjust their gestational lengths?

The mean total gestation length was 74.71 days (range 70–81 days) in pregnancies with an observed start (oestrus or mating). Though variation around this mean was modest (S.D. of 3.11 days or 4.2% of the mean), several factors were associated with gestation length (Table S7). Increases in group size (*β* = −1.08 [SD = 0.36], 95% CrI: [−1.81, −0.37], *p*_−_ = 1.00) and the body condition of mothers at the start of pregnancy were both associated with shorter gestations (*β* = −0.68 [SD = 0.39], 95% CrI: [−1.45, 0.09], *p*_−_ = 0.96), while increases in local primary vegetation productivity were associated with longer gestations (*β* = 0.64 [SD = 0.36], 95% CrI: [−0.06, 1.34], *p*_+_ = 0.96). Increases in temperature were also associated with shorter gestations, up to ca. 30°C, where the effect levelled off (Figure S11). Extended pregnancies arose primarily through elongating the period *before* the onset of GWG (73% of variation in gestation lengths, Figure 5) rather than delaying giving birth (27% of variation, *β* = 0.73 [SD = 0.33], 95% CrI: [0.08, 1.38], *p*_+_ = 0.98, Table S8). Extended pregnancies were associated with small reductions in the rate of GWG (*β* = −0.10 [SD = 0.60], 95% CrI: [−0.22, 0.02], *p*_−_ = 0.95). Gestation lengths were not associated with subsequent inter‐pregnancy intervals (*β* = −0.29 [SD = 0.06], 95% CrI: [−0.14, 0.09], *p*_−_ = 0.70).

### 3.4 How do gestational weight gains affect pup fates?

Pups born from pregnancies with high estimated *per capita* prenatal growth rates emerged from the burrow heavier (Figure 6b, Table S9, *β* = 3.55 [SD = 1.82], 95% CrI: [−0.01, 7.13], *p*_+_ = 0.97), but their telomeres were not shortened (Figure 6c, *β* = −0.002 [SD = 0.010], 95% CrI: [−0.02, 0.01], *p*_−_ = 0.62). Pups that emerged from the burrow heavier displayed higher survival to both nutritional independence (*β* = 1.06 [SD = 0.20], 95% CrI: [0.69, 1.46], *p*_+_ = 1.00) and adulthood (Figure 6d, *β* = 0.67 [SD = 0.14], 95% CrI: [0.40, 0.97], *p*_+_ = 1.00). There was no additional effect of *per capita* prenatal growth rates on pup survival after controlling for pup emergence body mass (Tables S11 and S12), indicating that the positive effect of rapid GWG on survival operated through increases in the body mass at emergence. Variation in the duration of GWG was not associated with the body mass or survival of emerging pups (Supporting Information S14). Pups born to older mothers emerged lighter and showed lower survivorship to independence and adulthood (see Supporting Information S15 and Tables S9–S12 for details of other covariates in models of pup body mass and survival).

## 4 DISCUSSION

We used repeated body mass measures from full‐term pregnancies to characterize the dynamics and consequences of GWG in a wild mammal for the first time. Our results suggest that under natural conditions, food availability limits GWGs, for growth was accelerated during periods of high local vegetation productivity and high temperatures, and mothers given supplemental food substantially increased GWGs. Variation in gestation length was small, limited to the early phases of pregnancies and primarily associated with prevailing environmental factors rather than reproductive competition. Faster GWG was an important predictor of offspring body mass after birth and survival to adulthood, and did not incur costs in terms of offspring telomere shortening.

Our analysis confirmed a striking biphasic pattern of GWG in meerkats, with negligible growth in the first 31 days, and linear growth in the following 44 days. Though biphasic GWGs are seen in other mammals, in meerkats the pattern is particularly pronounced, with a longer, flatter initial phase and a sharper increase in the growth phase (Long & Ebensperger, 2010; Rossner, 1997; Urison & Buffenstein, 1995). Poor primary productivity values were associated with a later onset of GWG, and extending the initial phase could therefore allow mothers facing unpredictable food availability to delay investment to limit resource losses in the event of abortion. The adaptive significance of variation in this biphasic pattern across species merits further investigation.

Our correlative results suggest that GWGs were more rapid during periods of high primary productivity, and our feeding experiment indicates that this is driven by food availability. Though less metabolically expensive than lactation, pregnancy incurs a high nutritional demand, driving an increased food intake of 45% in mice (Speakman, 2007) and an additional daily calorie demand of 1950 kJ in humans (Butte & King, 2005). Our results suggest that the arid and unpredictable Kalahari Desert imposes substantial nutritional constraints on pregnant meerkats: subordinate females were unable to accelerate their GWGs when carrying more pups, which likely explains why they typically produce litters with fewer pups compared to those of dominant females (MacLeod & Clutton‐Brock, 2013). Our experimental results suggest that even dominant females (who are in better condition than subordinates) are food‐limited, as they substantially accelerated their GWGs when fed. Broadly, our findings suggest the primary determinant of variation in GWGs is maternal nutrition during pregnancy.

We detected little variation in gestation lengths, and two results indicated that mothers have limited abilities to adaptively adjust birth timing. First, overall gestation lengths were shorter in larger groups and for heavier mothers, suggesting that mothers with stronger body condition and alloparental support may shorten gestations in order to quickly re‐conceive (Butte & King, 2005; Hill et al., 2000). However, shortened gestations were not associated with reduced inter‐pregnancy intervals, and mothers that gave birth earlier were thus able to capitalize on favourable conditions, but not to advance their subsequent reproductive schedules. Second, the duration of the growth phase of pregnancy did not respond to social conditions, despite extensive evidence that mammals can adjust their *postnatal* growth according to their social environment (Buston & Clutton‐Brock, 2022). Moreover, there is strong selection to delay birth during intense reproductive competition in meerkats, because early‐born litters are highly likely to suffer infanticide (Cram et al., 2019). Adaptations to delay birth, even under the imminent threat of infanticide, may therefore be impossible due to physiological or anatomical constraints (Gluckman & Hanson, 2004). Further work is needed to clarify how other mammals (including close relatives of the meerkat) adjust gestation lengths to optimize birth timing (Hodge et al., 2011) and the drivers of gestation length variance across species.

Adjustments to the *rate* of GWGs in response to social cues were similarly limited. Mothers did not invest more in their developing young when many females were breeding together, to prepare them for a competitive early environment (Meylan et al., 2007). By contrast, enhanced competition is associated with increased pre‐ and postnatal maternal investment in banded mongooses (*Mungos mungo*) and North American red squirrels (*Tamiasciurus hudsonicus*), respectively (Dantzer et al., 2013; Inzani et al., 2016). As outlined above, meerkats' harsh habitats may constrain maternal ability to accelerate foetal growth, even where it would be beneficial. Mothers also did not decrease their investment in developing young in larger groups, where they are likely to receive more alloparental care (‘concealed helper effects’; Dixit et al., 2017). Overall, while social cues appear to drive variation in postnatal growth in a variety of group‐living vertebrates (reviewed in Buston & Clutton‐Brock, 2022) including meerkats (Huchard et al., 2016), our results suggest that foetal growth plasticity in response to the social environment is more constrained.

Our results suggest that temperature strongly influences GWGs in income‐breeding mammals including meerkats, where foetal development relies on daily maternal foraging rather than stored energy reserves (Dalerum et al., 2007). Moderate warming within meerkats' thermoneutral zone (below 30–32°C; Müller & Lojewski, 1986) increased maternal foraging success leading to earlier inflection points, faster GWGs, and shorter gestations. At higher temperatures, however, mothers delayed investment and slowed GWGs, likely due to trade‐offs between foraging and heat‐avoidance behaviours (e.g. shade‐seeking, Van de Ven et al., 2020). Broadly, our results highlight that an income‐breeding strategy makes pregnant females particularly vulnerable to increasing temperatures, which force them to thermoregulate rather than forage, and thus compromise their ability to optimally invest in young (Beltrán et al., 2021).

Greater maternal investment in developing young typically increases their postnatal survival (Ronget et al., 2018; Vitikainen et al., 2023), and our results suggest that female meerkats achieve this by accelerating offspring prenatal growth. Pups born following faster GWGs were heavier post‐birth, and were consequently more likely to survive to nutritional independence and adulthood. Moreover, while rapid *postnatal* growth commonly shortens telomeres (McLennan et al., 2016; Ringsby et al., 2015), our results suggest that faster *prenatal* growth does not incur costs in this currency, which would likely select for mothers to maximize their investment in foetal growth. Notably, the survival benefits of faster GWGs appeared to be entirely mediated by improvements in birth weights, as these effects disappeared when we controlled for early life body mass. This suggests, first, that females with slow GWGs are unable to compensate through additional lactation, and second, that maternal body mass gained during pregnancy does not substantially contribute to the energetic costs of lactation (Butte & King, 2005), which must instead be met by post‐birth maternal foraging. In this species, there appear to be limited trade‐offs between energetic investment in gestating foetuses and subsequent lactation, as there are in other mammals (Barboza & Parker, 2008).

Overall, our analyses highlight the nutritional constraints imposed by harsh environmental conditions on pregnant females, and that failure to overcome these challenges can have marked effects on the resulting young. Further work is needed to clarify whether offspring can postnatally compensate for slow prenatal growth (and whether such ‘catch‐up’ growth incurs costs later in life; Kuijper et al., 2019), and how variation in GWG affects maternal health and survival. Our results also suggest that prenatal growth is not responsive to social cues, as we found limited adjustments to capitalize on the benefits or mitigate the challenges of the early‐life social environment offspring are likely to face. The lack of prenatal growth adjustments may increase selection for plasticity in postnatal growth, resulting in the pronounced strategic growth seen in many social species (Buston & Clutton‐Brock, 2022). Broadly, characterizing both the rate and duration of GWGs in wild mammals will improve our understanding of the constraints and adaptations governing maternal investment during pregnancy, and their consequences for offspring health and fitness.

## AUTHOR CONTRIBUTIONS

Jack Thorley: Data curation, analysis, investigation, methodology, software, validation, visualization, writing and editing; Tim Clutton‐Brock: Conceptualization, funding acquisition, investigation, project administration, resources and editing; Helen C. Spence‐Jones: Data curation, methodology, software, validation and editing; Zoe Turner: Data curation, methodology, software, validation and editing; Stuart P. Sharp: Conceptualization, investigation, methodology and editing; Marta B. Manser: Funding acquisition, project administration, resources and editing; Winnie Boner: Data curation, analysis, methodology, resources, software, validation and editing; Robert Gillespie: Data curation, analysis, methodology, resources, software, validation and editing; Dominic L. Cram: Conceptualization, data curation, analysis, investigation, methodology, project administration, software, validation, visualization, writing and editing.

## CONFLICT OF INTEREST STATEMENT

The authors declare no conflicts of interest.

## STATEMENT ON INCLUSION

This study was conducted in collaboration with South African stakeholders at the Kuruman River Reserve, South Africa, where the field site is located. The reserve supports the livelihoods of numerous South African people, including research and site managers, veterinarians, and operations and support staff. Data collection for this study was carried out by a large number of field interns from diverse national backgrounds, including some from South Africa and neighbouring countries, who received extensive training in field ecology. Their contributions are gratefully acknowledged but too numerous for individual co‐authorship. The Kalahari Meerkat Project continues to improve practices to embed research within local institutions and communities through capacity building, meaningful collaboration, and transparent acknowledgement of both contriubutions and challenges.

## Supporting information


**Figure S1.** The distribution of inter‐birth intervals (IBI) in meerkats.
**Figure S2.** The strong positive associations between conception mass and post‐birth mass in meerkats.
**Figure S3.** The data distribution for the maternal and social covariates, and the correlations among them, in pregnant meerkats.
**Figure S4.** Estimating body condition at the start of each pregnancy in wild meerkats.
**Figure S5.** The average body condition of all known‐age meerkats from 1998 to 2022.
**Figure S6.** Time series of the weekly mean maximum and minimum temperature, weekly total rainfall, normalized vegetation index (NDVI), and the average adult meerkat body condition at the Kalahari Meerkat Project, from 2002 to 2022.
**Figure S7.** The relationship between the age at first weighing (emergence) and body weight in wild meerkats.
**Figure S8.** The effect of dominance status, maternal age, and group size on variation in meerkat body weight at the start of pregnancy.
**Figure S9.** Effects of various predictors on the duration and rate of gestational weight gain in wild meerkats.
**Figure S10.** The duration of gestational weight gain was not associated with any fitness measure in wild meerkats.
**Figure S11.** The quadratic effect of temperature on gestation lengths in wild meerkats. Increases in temperature are associated with shorter gestation lengths, up until around 30ºC, after which point the average gestation length levels off.
**Table S1.** Within‐pregnancy correlations in the shape parameters of the gestational weight gain curve for wild meerkats.
**Table S2.** Models of gestational weight dynamics fitted in the study.
**Table S3.** Model 1 output.
**Table S4.** Model 2 output.
**Table S5.** Model 3 output: maternal, social, and environmental effects on variation in gestational body weight in wild pregnant meerkats.
**Table S6.** Model 4 output: The effect of supplemental feeding throughout pregnancy on gestational body weight in wild meerkats.
**Table S7.** Model output: factors affecting gestation length in wild meerkats.
**Table S8.** Model 5 output: body weight changes for meerkat pregnancies that started with an observed oestrus or mating event.
**Table S9.** The effect of gestational weight gain parameters on the body weight of meerkat pups at emergence from the birth burrow (approximately 18 days).
**Table S10.** The effect of gestational weight gain parameters on meerkat pup telomere length at emergence.
**Table S11.** The effect of gestational weight gain parameters on meerkat pup survival to independence (90 days).
**Table S12.** Model output: the effect of gestational weight gain parameters on meerkat pup survival to adulthood (1 year).

## Data Availability

Data and reproducible R code are available from the Dryad Digital Repository: https://doi.org/10.5061/dryad.j6q573nv5 (Thorley et al., 2025).
