# Supplementary material for: The ecology of gestational growth in a wild cooperative mammal
Source: J Anim Ecol. 2025 Dec 12;95(2):257–69. doi: 10.1111/1365-2656.70199 (PMC12868393; doi:10.1111/1365-2656.70199)
Supplement: Supplementary file 1 — Figure S1. The distribution of inter‐birth intervals (IBI) in meerkats. Figure S2. The strong positive associations between conception mass and post‐birth mass in meerkats. Figure S3. The data distribution for the maternal and social covariates, and the correlations among them, in pregnant meerkats. Figure S4. Estimating body condition at the start of each pregnancy in wild meerkats. Figure S5. The average body condition of all known‐age meerkats from 1998 to 2022. Figure S6. Time series of the weekly mean maximum and minimum temperature, weekly total rainfall, normalized vegetation index (NDVI), and the average adult meerkat body condition at the Kalahari Meerkat Project, from 2002 to 2022. Figure S7. The relationship between the age at first weighing (emergence) and body weight in wild meerkats. Figure S8. The effect of dominance status, maternal age, and group size on variation in meerkat body weight at the start of pregnancy. Figure S9. Effects of various predictors on the duration and rate of gestational weight gain in wild meerkats. Figure S10. The duration of gestational weight gain was not associated with any fitness measure in wild meerkats. Figure S11. The quadratic effect of temperature on gestation lengths in wild meerkats. Increases in temperature are associated with shorter gestation lengths, up until around 30ºC, after which point the average gestation length levels off. Table S1. Within‐pregnancy correlations in the shape parameters of the gestational weight gain curve for wild meerkats. Table S2. Models of gestational weight dynamics fitted in the study. Table S3. Model 1 output. Table S4. Model 2 output. Table S5. Model 3 output: maternal, social, and environmental effects on variation in gestational body weight in wild pregnant meerkats. Table S6. Model 4 output: The effect of supplemental feeding throughout pregnancy on gestational body weight in wild meerkats. Table S7. Model output: factors affecting gestation length in wild meerkats. Table S8. [file JANE-95-257-s001.docx]

**SUPPLEMENTARY MATERIALS**

**The ecology of** **gestational growth in a wild cooperative mammal**


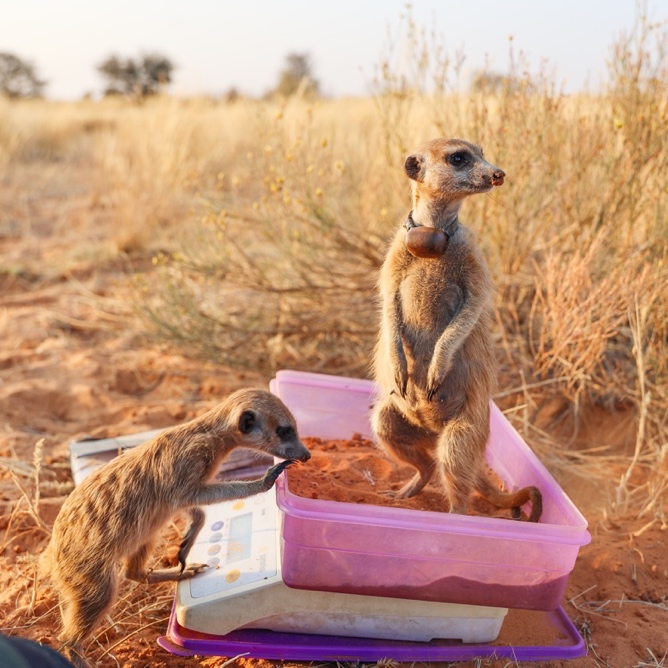


© Pam Hurkens, Kalahari Meerkat Project

| **Section** |  |  | **Pages** |
| --- | --- | --- | --- |
|  | **SUPPLEMENTARY METHODS** |  |  |
| §1 | Estimation of pregnancy length in meerkats |  | 1 |
| §2 | Hypotheses |  | 1-2 |
| §3 | Ultrasound determination of litter size |  | 2 |
| §4 | Modelling gestational body weight gain |  | 3-4 |
| §5 | Estimating female body condition at the start of pregnancy |  | 4-7 |
| §6 | Environmental covariates in relation to body condition |  | 7-8 |
| §7 | Effects of supplementary feeding on the duration and rate of gestational weight gain |  | 8-9 |
| §8 | Pup relative telomere length estimation: sampling and qPCR protocols |  | 9-10 |
| §9 | Standardising emergence weight for survival GLMs |  | 10-11 |
| §10 | How do gestational weight dynamics affect pup fates? |  | 11 |
|  | **SUPPLEMENTARY RESULTS** |  |  |
| §11 | The shape of gestational weight gain |  | 12 |
| §12 | Effects on maternal body weight at start of pregnancy |  | 13 |
| §13 | Effects on the duration and rate of gestational weight gain |  | 14 |
| §14 | Limited consequences of gestational weight gain duration on fitness |  | 15 |
| §15 | Supplementary tables: model structures and outputs |  | 16-31 |

**SUPPLEMENTARY METHODS**

**§1 Estimation of pregnancy length in meerkats**

Meerkat pregnancies are estimated to be 70-75 days in length, because females (i) have inter-birth intervals (IBI) that can be as short as 74 days in the wild (Doolan & Macdonald, 1997) and (ii) experience a post-partum oestrus lasting approximately one week (Moss et al., 2001), during which time dominant males will guard and mate with the receptive female (Kutsukake & Clutton-Brock, 2007). In our population, the modal IBI was 79 days (n = 85 occasions where IBI could be precisely determined), with a lower bound of 76 days (Figure S1). If conception occurs within the first week post-partum, this suggests a lower bound on pregnancy of 69-75 days and a modal length of 72-79 days. The upper bound on pregnancy length is less clear. Recent work in our study population suggested that females can prolong gestation by ca. 3.3 days when aggressively evicted from their natal group (Maag et al., 2023). Since our dataset does not include females that were evicted during their pregnancy, we assumed that each pregnancy was 75 days in length (the centre of the modal length estimate), working backwards from the birth date.


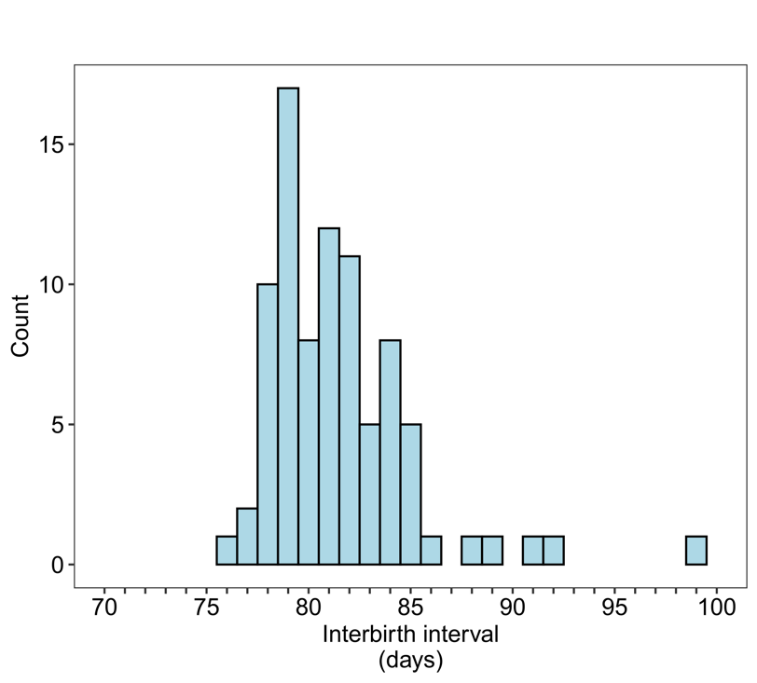


**Figure S1**. **The distribution of inter-birth intervals (IBI) in meerkats. To be included in this data subset, two concurrent pregnancies from the same female had to be successful (the pups were carried to term), and the date of birth had to be known exactly in each case: all inter-birth intervals are known exactly. The right-hand side of the distribution was limited to 100 days as gestations are highly unlikely to be extended by ca. 50%. The data reveal that IBIs cannot be shorter than 76 days and are most commonly 79 days.**

**§2 We tested the hypothesis that gestational weight gains tightly reflect foetal growth in wild meerkats.**

This hypothesis predicts that:

1. The majority of weight gained during pregnancy is lost during parturition.
   - Our data shows that a female’s body mass immediately after giving birth closely matches her mass at conception (Figure S2). This tight, near 1:1 correlation suggests that little of the body mass gained during pregnancy is retained after birth.
2. Meerkats do not accumulate weight during gestation that is stored until after birth and used for lactation.
   - Previous isotopic analysis of lactating female meerkats suggested that they are income breeders rather than capital breeders: “small body size coupled with a high selection pressure to remain agile to escape predators prevents meerkats from building body reserves” (Dalerum et al., 2007).
3. Weight gained during pregnancy strongly predicts pup emergence weight.
   - Our analyses reveal a strong positive effect of GWG on pup emergence weights (Figure 6, Table S9, β = 3.55 [SD = 1.82], 95% Credible Intervals (CrI): [-0.01, 7.13], p_+_ = 0.97).


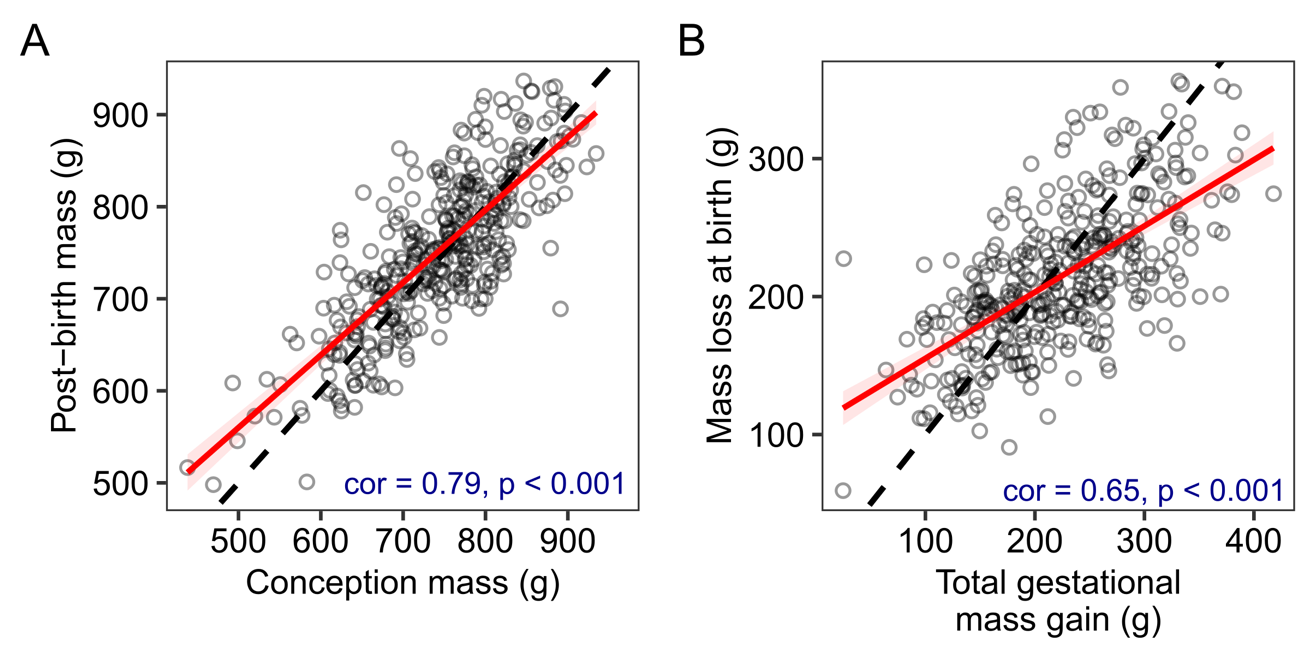


**Figure S2. The strong positive associations between conception mass and post-birth mass in meerkats. Conception mass values were estimated from the pregnancy trajectories; post-birth mass was taken as the mean mass in the week after pregnancy.**

**§3 Ultrasound determination of litter size**

We checked that emerging litter size accurately reflects *in utero* litter size by counting developing fetuses using a Sonoscape S6 ultrasound scanner and L742 linear probe (Sonologic, Shenzhen, China) during routine captures of females that were not visibly pregnant. We gently picked up a female meerkat by the tail base, carefully placed them into a cotton sack and anaesthetised them with isoflurane (Isofor; Safe Line Pharmaceuticals, Johannesburg, South Africa) in oxygen, using a vehicle-mounted vaporiser (Jordan et al. 2007). We scanned sedated females by passing the linear probe over the abdomen and counted fluid-filled pregnancy sacs (which each contain one fetus). Of 29 scans of 29 pregnant females, 12 litters (41.4%) were later entirely lost to infanticide. In the remaining 17 litters, 68 pups emerged out of 69 detected embryos, a loss of only 0.06 ± 0.56 (mean ± standard deviation (S.D.)) pups per litter. Partial litter loss is therefore exceptionally rare and emerging litter size is an accurate indicator of *in utero* litter size (Inzani et al., 2016; Russell et al., 2002).

**§4 Modelling gestational body weight gain**

In Model 1, we characterised the shape of gestational weight gains across all pregnancies. If $y_{ij}$ is the measured body weight of a female for the *i*th pregnancy at time $t_{ij}$, with *j* being the day of pregnancy, then:

$$y_{ij} \sim N\left( \mu_{ij,}\sigma_{y}^{2} \right)$$

$\mu_{ij}= \beta_{1i}+ \beta_{2i}t_{ij}.I\left( {\omega_{i} \leq t}_{ij} \right)+\left( \beta_{2i}\omega_{i}+ \beta_{3i}\left( t_{ij}- \omega_{i} \right) \right).I\left( t_{ij} > \omega_{i} \right)$,

where $\beta_{1i}$ is the pregnancy-specific intercept (body weight at the start of pregnancy), $\beta_{2i}$ is the pregnancy-specific pre-inflection slope, $\omega_{i}$ is the pregnancy-specific inflection point (onset of gestational weight gain), and $\beta_{3i}$ is the pregnancy-specific post-inflection slope (rate of gestational weight gain). I(.) is then a logical indicator function which toggles ‘on’ or ‘off’ according to whether the day of pregnancy falls before or after the inflection point, with the post-inflection slope being estimated relative to body weight at the inflection point, $\beta_{2i}\omega_{i}$. The pregnancy-specific effects $\beta_{1i}$, $\beta_{2i}$, $\beta_{3i}$, and $\omega_{i}$ are then further specified as:

$$\left[ \begin{matrix} \beta_{1i} \\ \beta_{2i} \\ \beta_{3i} \\ \omega_{i} \end{matrix} \right]= \left[ \begin{matrix} \beta_{10} \\ \beta_{20} \\ \beta_{30} \\ \omega_{0} \end{matrix} \right]+ \left[ \begin{matrix} u_{1i} \\ u_{2i} \\ u_{3i} \\ u_{4i} \end{matrix} \right]$$

where $\beta_{10}$, $\beta_{20}$, $\beta_{30}$, and $\omega_{0}$ are the population-level average intercept at the start of gestation, the pre-inflection slope, the post-inflection slope, and the inflection point, respectively (fixed effects; hereafter *shape parameters*), and $u_{1i}$to $u_{4i}$ denote the pregnancy-specific deviations from the population average (random effects). We also included an effect of litter size on both the inflection point and the post-inflection slope, because of the prior expectation that litter size would influence the rate and possibly also the onset of gestational weight gain. The model also estimated the correlations among the pregnancy-level random effects - among $u_{1i}$, $u_{3i}$, and $u_{4i}$ specifically - to examine whether the different aspects of the gestational growth curve were correlated, after controlling for litter size. For example, if heavier females tend to invest more in gestational weight gains, then this would result in a positive correlation between $u_{1i}$ and $u_{3i}$. In Model 2, we included random effects of female identity and breeding season for each of the shape parameters, to examine whether pregnancy-level correlations among the shape parameters changed after controlling for these random terms. Breeding seasons were defined from July 1^st^ to June 30^th^ to capture the seasonal changes in meerkat reproduction in the Kalahari. The estimated inflection point can be converted into the duration of gestational weight gains by subtracting it from 75. Equally, covariate effects on inflection timing (see below) can be translated into effects on gestational weight gain duration by multiplying their estimates by -1.

We extended Model 2 to examine whether maternal, social, and environmental factors were associated with variation in gestational body weight (Model 3). Specifically, we examined whether the covariates of interest affected the body weight at the start of gestation (*β*_10_), the timing of the onset of gestational weight gain (*ω*_0_, the inflection point), and the rate of gestational weight gains (*β*_30_, the post-inflection slope).

Unless otherwise stated, all model sampling proceeded with four chains of 5000 iterations, of which 2000 were dedicated to the warm-up. We used informative priors for the shape parameters ($\beta_{10}$, $\beta_{20}$, $\beta_{30}$, $\omega_{0}$) guided by the observed phenotypic variation in the raw data (Figure 1). For example, the prior on $\beta_{10}$ was based on the average weight of mothers at the start of gestation (normal(mean = 750, sd =100)), while priors for the slope parameters $\beta_{20}$ and $\beta_{30}$ reflected typical daily weight gain rates observed before and after the inflection point. The prior on the inflection point $\omega_{0}$ was informed by the biphasic shape common to most pregnancies (normal(35, 10)). In contrast, predictor variables - factors affecting the shape parameters - were given weakly informative priors centred on zero and with standard deviations reflecting the magnitude of the variation in the shape parameter: typically, for $\beta_{10}$, normal(0, 20); for $\beta_{30}$, normal(0, 10); and for $\omega_{0}$, normal(0, 2),. All continuous predictors displayed low collinearity (Figure S3, all variance inflation factors < 1.24). Model checks indicated adequate mixing of chains and a close correspondence between the observed data and posterior predictive distributions throughout.


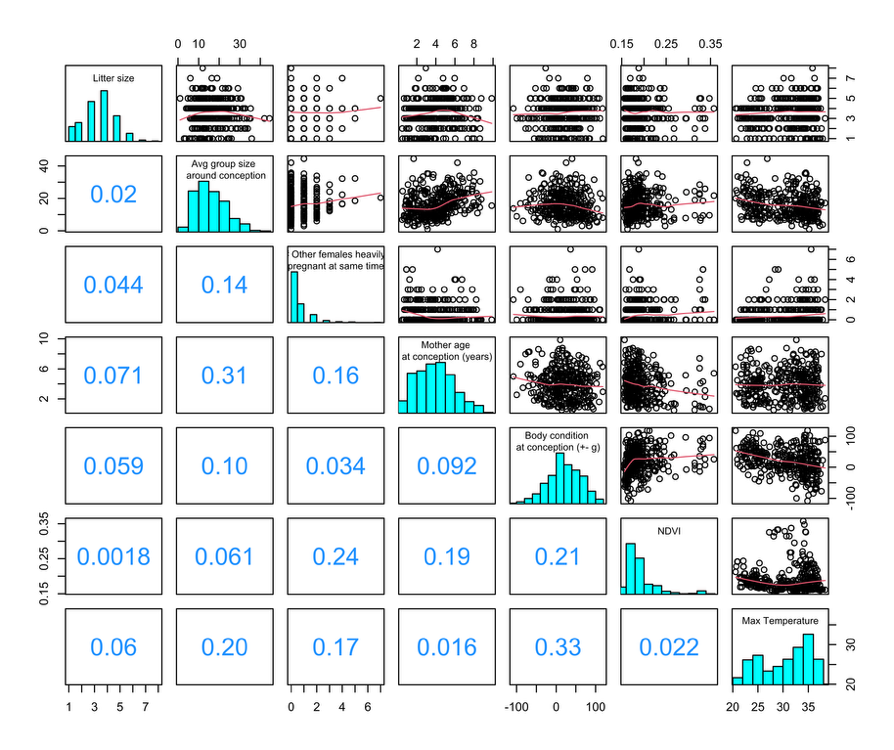


**Figure S3. The data distribution for the maternal and social covariates, and the correlations among them, in pregnant meerkats. Each point is unique to a single pregnancy (n = 381). The upper triangle of the matrix displays raw data correlations with a loess smoother fitted, the lower triangle displays the spearman’s rank correlation, and the diagonal displays the frequency distribution. Max temperature here is the mean maximum temperature across pregnancy (whereas it was modelled as the mean temperature in the first or second half of pregnancy).**

**§5** **Estimating female body condition at the start of pregnancy**

To estimate female body condition at the start of pregnancy, we modelled the lifetime growth curves of all known-age individuals in our study population. Growth was modelled as a single non-linear mixed effects model, with body condition then estimated from the age-specific residuals of this model as described below. The model took the form of a biphasic monomolecular curve:

$$W= \left\{ \begin{aligned} A\left( 1- e^{-k_{0}\left( t-t_{0} \right)} \right) ;for t< t_{1} \\ A\left( 1- e^{-k_{0}\left( t-t_{0} \right){-k}_{1}\left( t-t_{1} \right)} \right) ;for t\geq t_{1} \end{aligned} \right.$$

where *W* is body weight at time *t*, *A* is the asymptotic body weight, *k_0_* and *k_1_* are growth rate constants, and *t_0_* is the age at onset of growth. *t_1_* is then the threshold age at which the growth rate constant changes from *k_0_* changes to *k_1_*, which we set at 90 days. A, *k_0_*, *k_1_*_,_ *t_0_* were all allowed to vary as uncorrelated, individual-level random effects.

This two-phase model, first outlined by English et al. (2012), captures the decrease in the growth rate of meerkats as they become nutritionally independent of helpers at 90 days of age, and conforms to the concave shape of meerkat growth following emergence from the natal burrow at approximately 2-3 weeks of age. The model accurately predicts individual variation in body weight across lifespan (Figure S4) and can be further extended to allow for seasonal effects on growth (an alternative parameterization of the monomolecular growth model can be seen in Veylit et al., (2021). We do not include any seasonal effects here as we were interested in the residual body weight of individuals independent of the environment: which we take to represent body condition at any given point across lifespan (the relative amount of ‘on-body’ reserves).

We fitted the model to 667,643 body weight measurements taken from 3,248 individuals as they emerged from their sleeping burrow in the morning, prior to commencing foraging. The data set covered the period from December 1996 to February 2023, and all known-age individuals that had been weighed at least 10 times were included. On average, individuals were weighed every 16.26 days across their lifespan (median = 3.05, 1SD = 106.49). We excluded any periods when females were pregnant but otherwise included all individuals in our study population (rather than just those females appearing in the pregnancy dataset) so that all available data was used to estimate individual growth curves. Males and females are monomorphic so can be modelled together. The partial pooling (shrinkage) of mixed effects models then ensures that individual-level estimates are shrunk towards the global mean over the entire dataset. This means that individuals with relatively fewer data ‘borrow’ information from individuals with relatively more data.

Figure S4 displays the biphasic model fit for three random individuals across lifespan, in addition to three further long-lived [dominant] females who were pregnant repeatedly and who therefore appear in our models of gestational weight gain. In each case the solid line displays an individual’s age-specific weight across their lifespan. Each point is a single body weight measurement, which is coloured according to its deviation from the predicted age-specific body weight: positive deviations reflect times when females were in relatively good body condition, while negative deviations reflected periods of poorer body condition. As noted above, weight measurements taken during pregnancy were excluded from modelling but are overlain here to highlight pregnancies (unfilled orange).

The body condition of females at the start of pregnancy was then estimated as the average residual body weight in the two-week period prior to the start of each pregnancy (i.e., 90-76 days prior to parturition). This can be visualized by zooming in on the body weight curve of female ‘*meerkat 7’* between the ages of four and six: shown in the lower panel below (B). Over this period, this dominant female was pregnant six times - vertical shaded rectangles - and her body condition across these pregnancies varied from relatively good (e.g., pregnancy 3) to relatively poor (e.g., pregnancy 6). If a female was not weighed within the two-week window, then the pregnancy was excluded from any models of gestational body weight change that included body condition as a covariate.


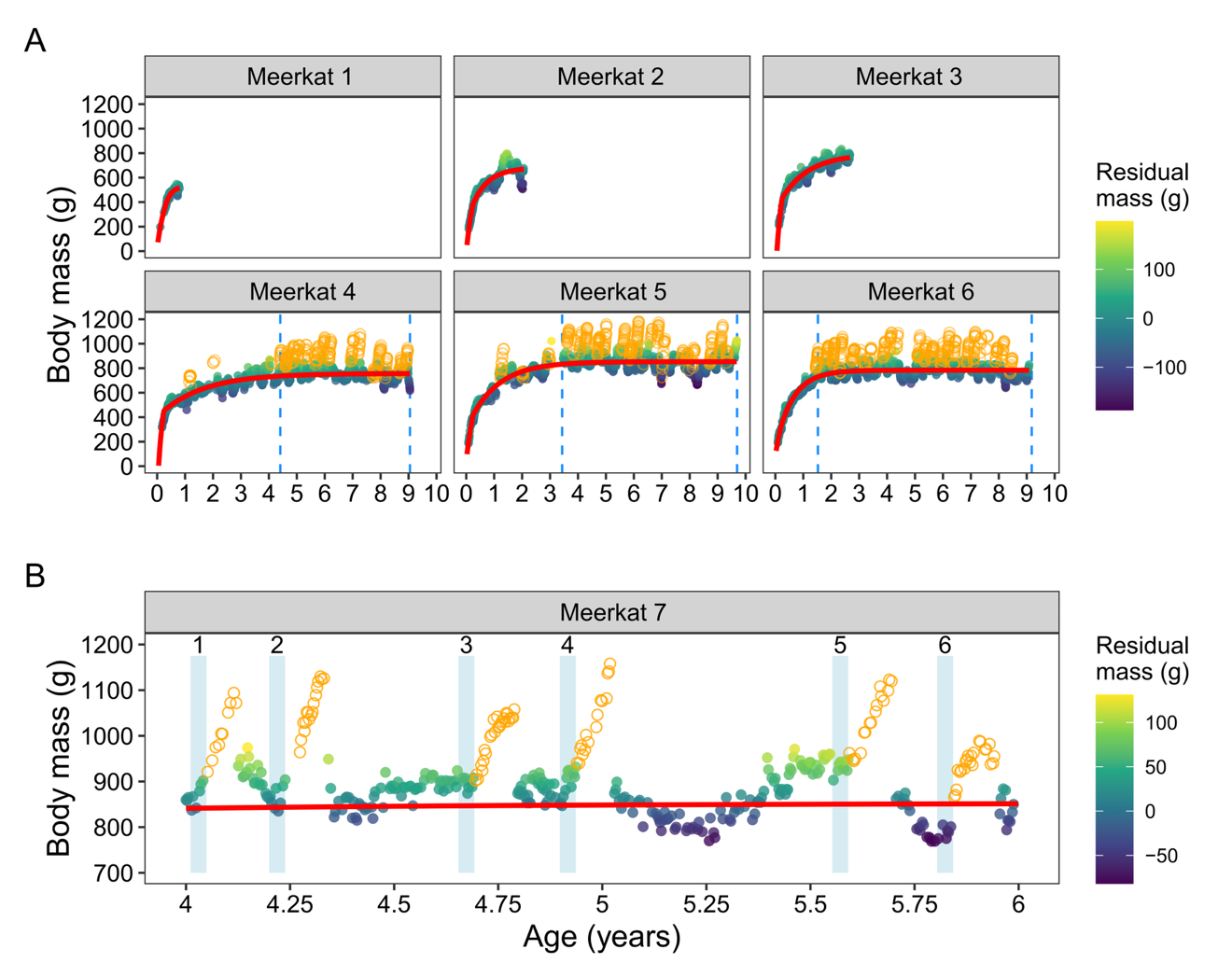


**Figure S4. Estimating body condition at the start of each pregnancy in wild meerkats. The predicted body weight of six meerkats across lifespan is shown in the upper two panels (A). Individual growth curves were derived from a biphasic monomolecular function fitted as a non-linear mixed effects model (NLMM) to body weight data collected outside of pregnancy. Here, each panel displays the predicted body weight for three random individuals who died before acquiring dominance, as well as three dominant females with especially long tenures. The raw body weight data is coloured according to the deviation of a given body weight measurement from the predicted age-specific body weight for an individual. Weight measurements taken during pregnancy were not fitted to models, but are highlighted in orange (unfilled points). The start and end of each female’s tenure is shown by the two vertical dashed lines. Using these residuals, the body condition of a female at the start of her pregnancy was estimated as the average residual body weight two weeks prior to the start of pregnancy. (B) This two-week window is shown for six pregnancies of female ‘meerkat 7’ when she was between four and six years of age (blue rectangles).**

By plotting the residual weight of all individuals in our study population as a time series from 1998 to 2022 (also in Thorley et al. 2025a), we can also see that that our growth modelling approach is able to capture general changes in the body condition of all individuals in the study population as environmental conditions change (Figure S5). For example, a drought period at the end of 2015 is reflected in a large drop in the average condition of most individuals in the study population. Body condition then rebounds again in early 2016 after a period of rainfall. The general trend in body condition is shown as a weekly moving average of the residuals (red trend line).


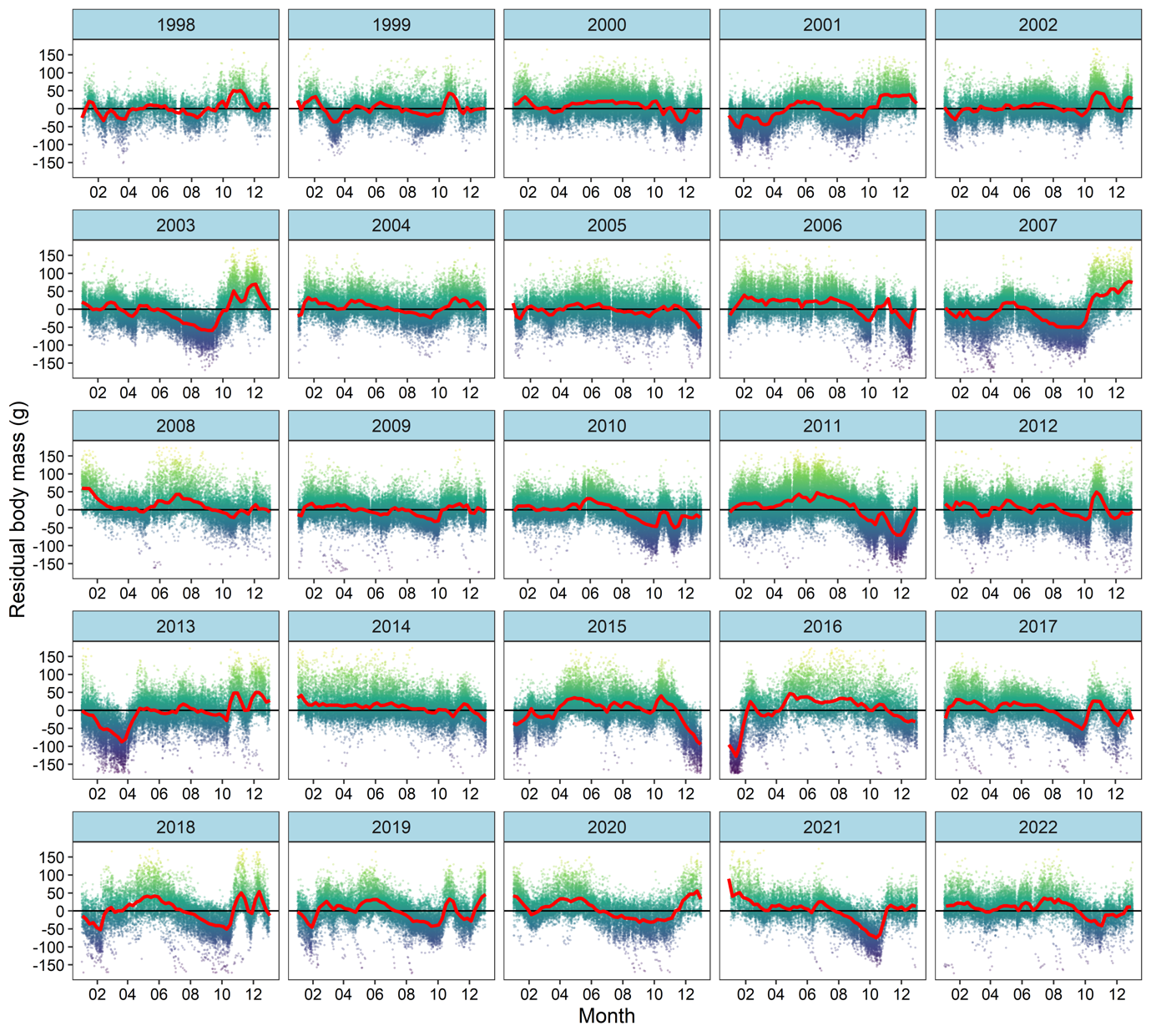


**Figure S5. The average body condition of all known-age meerkats from 1998 to 2022. Each panel displays the residual body condition of all individuals in the study population, with each point providing the relative deviation of a single body weight measurement from an individual’s long-term body weight trajectory (NLMM). The red line provides a moving average of the weekly residual weight of all individuals that were weighed within a given week.**

**§6 Environmental covariates and their relation to body condition**

We extracted daily air temperatures for our field site from NOAA’s CPC product. These data are provided on a 0.5-degree longitude-latitude grid and are highly correlated with time-matched values from the weather station at our field site. Temperature was taken as the mean daily maximum air temperature in the first (for the inflection point) or second (for the post-inflection slope) half of pregnancy.

Primary productivity was taken as the mean normalised difference vegetation index (NDVI) across the population’s range in the first half of pregnancy. NDVI in the Kalahari is strongly predicted by the total rain falling in the previous 1 or 2 months. Increases in NDVI have been related to increases in the abundance of the invertebrate prey of meerkats over short time scales, which positively affects meerkat body weight in turn (Thorley *et al.*, 2025a). We thus opted to use NDVI because it is more directly related to variation in food availability than rainfall is. The MODIS product only began recording in February 2000 (MODIS MOD13Q1), so Model 4 was restricted to 355 pregnancies that occurred after this point.

NDVI was extracted from the MODIS MOD13Q1 product and processed with the MODISTools package (Hufkens, 2022). These data are provided every 16 days on a 250-metre grid and in recent analyses have been shown to positively influence meerkat body weight over periods of 1-3 weeks (Thorley et al., 2025a). We restricted the NDVI data to areas of active space use following Thorley et al. (2025b). Briefly, NDVI for each 16-day period was calculated as the mean NDVI across all pixels that overlapped with the home ranges of frequently visited meerkat groups


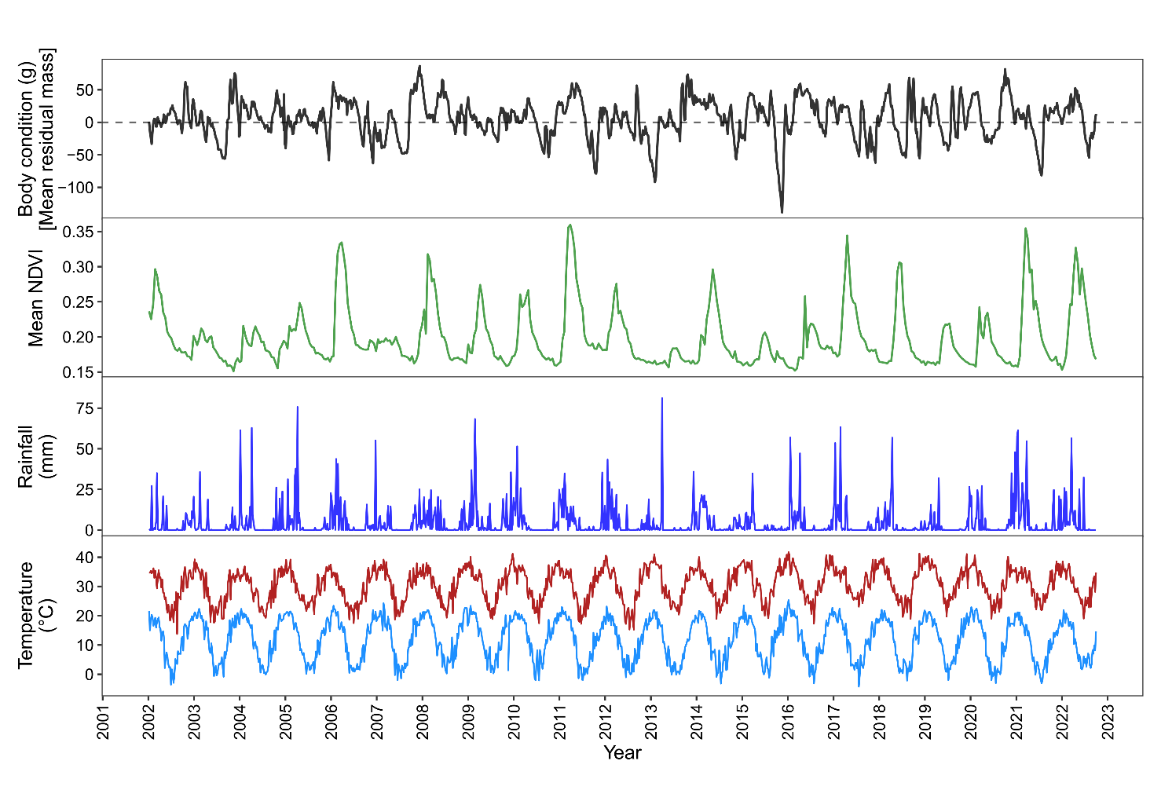


**Figure S6. Time series of the weekly mean maximum and minimum temperature, weekly total rainfall, normalized vegetation index (NDVI), and the average adult meerkat body condition at the Kalahari Meerkat Project, from 2002-2022. Overall, the figure demonstrates that upturns in NDVI are usually associated with an increase in rainfall over short time lags. Increases in rainfall/NDVI also lead to short-term increases in body condition. It is likely that climatic conditions and other aspects of phenology (such as the seasonal availability of certain prey types), demography (population density), and life history (such as the prevalence of TB) interact in complex ways to affect body condition.**

**§7 How does supplementary feeding affect the duration and rate of gestational weight gain?**

Between August and November 2011, ten dominant females were randomly assigned to either the ‘fed’ or ‘unfed’ (control) treatments, and where possible they were given the reverse treatment in the next breeding season (commencing between August and November 2012). While this protocol aimed to generate a fully factorial design in which all females received both treatments, some litters were lost and could not be included in our analyses. In addition, one female was inadvertently given the ‘fed’ treatment in two pregnancies. Exclusion of her second ‘fed’ pregnancy did not qualitatively affect the results. The final dataset included nine ‘fed’ pregnancies, four of which were matched with an ‘unfed’ pregnancy from the same mother. We selected five additional natural (‘unfed’) pregnancies from dominant females in the same population in the same breeding seasons. The birth dates of pregnancies in the two treatments fell within 36.9 ± 25.0 days of each other (mean ± 1SD, range = 2 to 83), and there was no difference between treatments in the body condition of females at the start of the pregnancy (t-test, t = 0.33, df = 10.1, p = 0.75), or their resultant litter sizes (t-test, t = 0.67, df = 14.4, p = 0.52).

**§8 Pup relative telomere length estimation: sampling and qPCR protocols**

Shortly after the litter's first emergence, a small biopsy of skin from the tail tip was collected from each pup (age 28.3 ± 3.4 days) for the determination of telomere length and parentage (Griffin et al. 2003; Spong et al., 2008). Skin samples were immediately transferred to 96% ethanol and stored at −20°C until DNA extraction.

For DNA extraction, 25mg of skin was excised from each tail tip, mixed with 180μl MACHEREY-NAGEL Lysis Buffer T1 and 25μl Proteinase K (20mg/ml) (MACHEREY- NAGEL GmbH & Co. KG, Düren, Germany), and incubated at 56°C for 1-3 hours until the tissue was completely lysed. DNA was then purified from lysate using MACHEREY-NAGEL NucleoMag® Blood 200μL kits (MACHEREY-NAGEL GmbH & Co. KG, Düren, Germany) in tandem with KingFisherTM Flex Purification System (Thermo Scientific, Wilmington DE, USA), following kit protocols. DNA was eluted in kit elution buffer MBL5 (5 mM Tris, pH 8.5) and stored at -20°C until further use. DNA concentration and purity were assessed using a Nanodrop-8000 Spectrophotometer (Thermo Scientific, Wilmington DE, USA). Average DNA concentration was 135.2 ± 83.8 ng/ul (mean ± standard deviation (S.D.)) and average 260/280 ratio was 1.98 ± 0.08. DNA integrity was assessed by running 10ng of DNA in a 0.8% agarose gel and was deemed to be acceptable for telomere measurement.

We used quantitative PCR (qPCR) analysis to measure telomere length, based on published protocols, with some modifications (Cawthorn et al., 2003; Criscuolo et al., 2009). This measure represents the average telomere length across cells in a sample, and is reported as the level of telomeric sequence abundance relative to a reference non-variable copy number gene. For qPCR measurement of relative telomere lengths, we used RAG1 as a reference gene due to its known status as a single copy gene in vertebrates (Crottini et al., 2012), and used a primer pair designed from Accession JQ073171.1 selected for good performance, lack of non-specific binding, and lack of primer-dimer (confirmed by melt curve analysis and gel electrophoresis) during optimization. HPLC purified primers were synthesized by IDT® (Integrated DNA Technologies, Leuven, Belgium) resuspended, diluted and stored at -20°C until assays were run. DNA samples (1.25ng) were assayed in triplicate and on separate plates for telomere and single-copy targets. Reactions were conducted using 1X Absolute blue qPCR SYBR green Low Rox master mix (Thermo Scientific, Wilmington DE, USA) with RAG1 forward (5’-CAT TGA GAC AGT CCC TTC CAT AG-3’) and reverse (5’- GGA GGC ATT GGG ATT CTT GTA-3’) primers at 500nM and telomere primers Tel1b (5’- CGG TTT GTT TGG GTT TGG GTT TGG GTT TGG GTT TGG GTT-3’) and Tel2b (5’- GGC TTG CCT TAC CCT TAC CCT TAC CCT TAC CCT TAC CCT-3’) (Epel et al., 2004) at 900nM, bringing reaction volumes up to 25μl with water. Mx3000P 96-well skirted plates (Agilent, Santa Clara, United States) were manually loaded, sealed with 8x strip optical caps (Agilent, Santa Clara, United States) and run in an Agilent Technologies Stratagene Mx3005P real-time PCR machine. RAG1 thermal profile was 15 min at 95°C, followed by 40 cycles of 15 s at 95°C, 30 s at 60°C, 30 s at 72°C. Telomere thermal profile was 15 min at 95°C, followed by 30 cycles of 15 s at 95°C and 30 s at 58°C. Both assays were followed by melt curve analysis of (58–95°C 1°C/5 s ramp). Dissociation curves showed a single peak for both assays in all reactions. The telomere assay had occasional very late amplification in the no template control (NTC) (Cq<28; except for one reaction amplifying at Cq>25). This was deemed to be late formation of primer-dimer and acceptable considering the highest Cq of our DNA samples (16.18). Following optimization, the RAG1 assay showed late amplification (Cq>35) in some reactions for the NTC, deemed to be primer-dimer formation. Comparing dissociation curves NTC with experimental samples showed much lower melting temperature and fluorescence in the NTC. With no sign of this peak profile in experimental samples and with a Cq much later than the highest of our samples (27.91), this was deemed to be acceptable.

A pooled aliquot of 33 DNA samples was serially diluted (5ng to 0.31ng) to generate a 5-point standard curve for each plate, which was used to calculate plate efficiencies (1.947-2.033 for RAG1 plates; 1.964-2.137 for telomere plates). The R2 for all plates was >0.985. Quantification cycles (Cqs) were imported into qBase + V3.0 (Biogazelle, Zwijnaarde, Belgium) for further calculation. Based on the ΔΔCq method, qBase+ calculates relative quantities of a sample (RQ) for each target by comparing Cqs of a sample with the average Cqs of all samples for that gene, controlling for reaction efficiency of that particular plate. RQ is then normalized by dividing by the geometric mean Cq of the reference gene, giving normalized relative quantity (NRQ). We used an inter-run calibration step with three samples run on every plate as inter-run calibrators (2.5ng and 0.625ng standard points and one “golden” sample run on every plate). This calculates calibrated normalized relative quantities (CNRQ) of telomeric DNA, equivalent to T/S ratios (Hellemans et al., 2007). Technical replicates falling outside 0.5Cqs were excluded or repeated, and samples were assigned to plates randomly. The average intraplate coefficient of variation of the Cq values for RAG1 and telomere plates were 0.18% and 0.68%, respectively. Intraplate variation was calculated as the coefficient of variation of the 1.25ng standard curve sample within a given plate. Interplate variation, calculated as the coefficient of variation of the ΔCq for the 1.25ng standard curve sample between plates was 3.37%, and coefficient of variation of the CNRQ (T/S ratio) of the 1.25ng standard curve sample across plates was 11.24%.

**§9 Standardising emergence weight for survival GLMs**

Pups that were weighed for the first time at a relatively younger age were generally lighter than pups weighed later (Figure S6A, Table S9). To control for this age-related variation in models of pup survival - where we wanted to include emergence weight as a predictor - we standardised the first weight of each pup by estimating their predicted weight at 25 days (the mean age at first weight = 24.8 days).

To do so, we regressed body weight against age for all weights taken from pups between 12 and 30 days of age across the history of our project (n = 6711 at time of analysis). This regression indicated that on average, individuals increased their weight by approximately 2.08g/day during this period, with growth then accelerating shortly afterwards (not displayed).

Based on this regression, we shifted the emergence weight of each pup according to the population-level slope (Figure S6B), either increasing or decreasing the weight according to whether they were weighed before or after 25 days of age. For example, the blue individual in Figure S6B was first weighed at 61g on 12 days of age. Under the expectation that individuals gain approximately 2.08g/day, we estimated that this pup would have gained 27g by the time it was 25 days of age and therefore weigh 88g. This process was applied to all pups and this corrected “emergence weight” was included in subsequent models of survival.


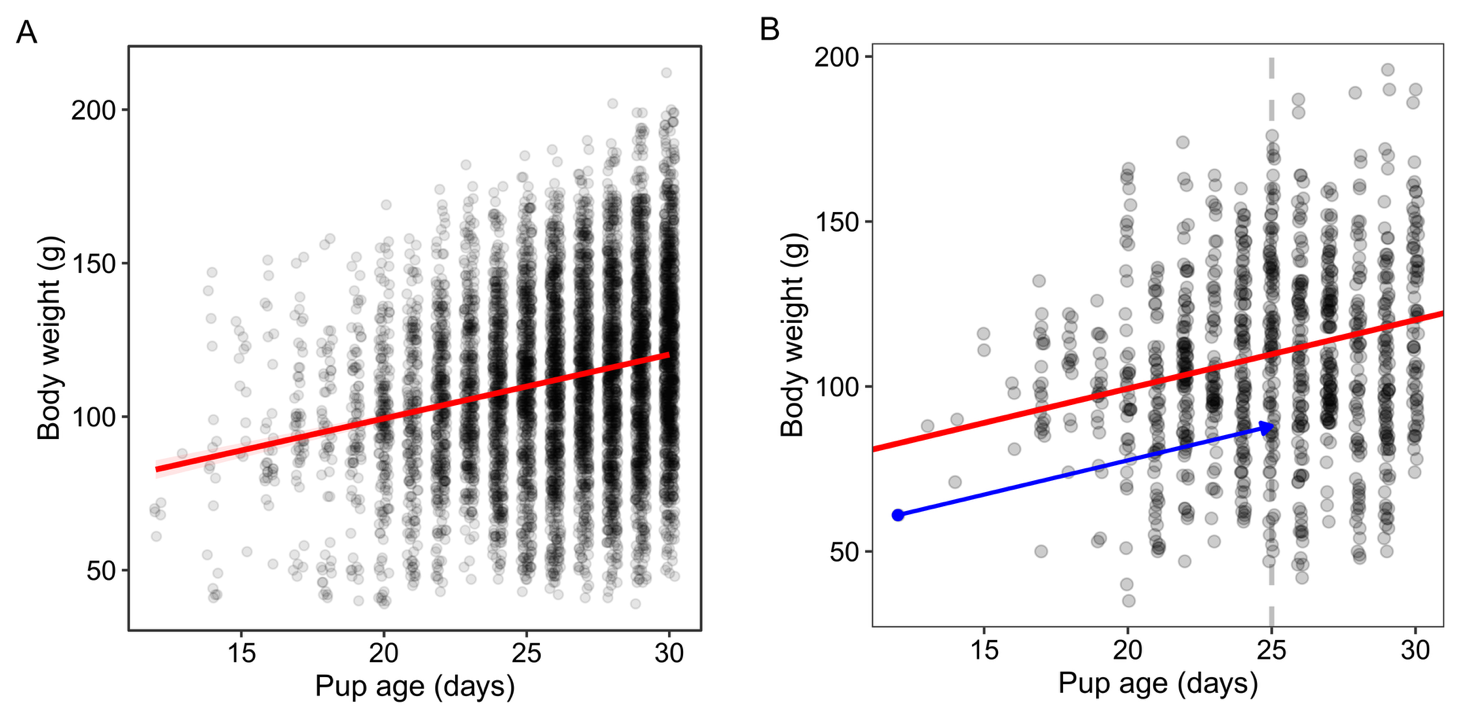


**Figure S7. The relationship between the age at first weighing (emergence) and body weight in wild meerkats. The linear regression between age and weight for all weights in our database (A). Given the predicted slope of 2.09g/day between 12 and 30 days of age, we standardized the emergence weight record for every individual (B) to their estimated weight at 25 days by shifting the record according to the population level slope. The blue arrow represents this adjustment for the lightest individual in the data set.**

**§10 How do gestational weight dynamics affect pup fates?**

To account for uncertainty in parameter estimation for the growth duration and post-inflection slope for each pregnancy, we drew 100 random samples from the posterior distribution of the initial model for each model parameter. In practice, this meant sampling the posterior distributions of 100 random iterations of the model, drawing evenly across the four chains (25 draws/parameter/chain). This produced 100 separate datasets, each of which contained a unique estimate for the duration and rate of weight gain for every pregnancy, as well as population-level estimates for each metric. The population-level estimates were added to the pregnancy-level estimates to yield the predicted duration and rate of gestational weight gain of each pregnancy. We then modelled each of the 100 distinct datasets individually and combined the posterior estimates across all sub-models (via the *brm_multiple* function). To reduce the computational running time and limit the size of the model output, we sampled two chains of 2000 iterations for each sub-model, 1000 of which were dedicated to the warm-up; and set a thinning interval of two (leading to 1000 posterior samples/sub-model).

**SUPPLEMENTARY RESULTS**

**§11 The shape of gestational weight gain**

There was striking variation in the shape of gestational weight gain across pregnancies, across individual mothers, and across breeding seasons (Tables S2-S3), as well as correlations among the shape parameters (main text Figure 1; Table S1). After controlling for the size of the litter, there was a weak positive correlation between the body weight of a female at the start of her pregnancy and the pregnancy’s inflection point (Model 1, corr = 0.10 [SD = 0.06], 95% CrI: [-0.02, 0.22], p_+_ = 0.95), and between the inflection point and the rate of gestational weight gain (Model 1, corr = 0.30 [SD = 0.06], 95% CrI: [0.18, 0.41], p_+_ = 1.00). This suggests that relatively heavier females tended to have shorter periods of gestational weight gain, and shorter periods of gestational weight gain tended to coincide with faster growth rates. However, the former correlations lost statistical support after the inclusion of random effects for the identity of the mother and the breeding season (Model 2, corr = 0.07 [SD = 0.08], 95% CrI: [-0.08, 0.21], p_+_ = 0.82), and there sign of the correlation between initial body weight and the gestational growth rate became negative (Model 2, corr = -0.06 [SD = 0.07], 95% CrI: [-0.19, 0.07], p_-_ = 0.82). As initial body weight in this case is corrected for the identity of the mother, this suggests that when mothers are in relatively better condition, their pregnancies have lower gestational growth rates, which is supported by further analyses below. The positive correlation between the inflection point and the rate of gestational growth at the level of pregnancy was retained in the model with all random effects included (Model 2, corr = 0.35 [SD = 0.06], 95% CrI: [0.22, 0.46], p_+_ = 1.00).

**Table S1. Within-pregnancy correlations in the shape parameters of the gestational weight gain curve for wild meerkats.** Estimates refer specifically to the correlation between random effects at the pregnancy level (i.e., among $u_{1i}$, $u_{3i}$, and $u_{4i}$) for our two models: controlling for the effect of litter size on the inflection point and the post-inflection slope (Model 1), and controlling for the effect of litter size, female identity and breeding season on each of the shape parameters (Model 2).

| ***Parameter*** | ***Posterior mean [L95%CrI, U95%CrI]*** | |
| --- | --- | --- |
|  | ***+ litter size*** | ***+ litter size, female ID, & breeding season*** |
| *corr* Initial body weight - inflection point | 0.10 [-0.02, 0.22] | 0.07 [-0.08, 0.21] |
| *corr* Initial body weight - post-inflection slope | 0.04 [-0.06, 0.15] | -0.06 [-0.19, 0.07] |
| *corr* Inflection point - post-inflection slope | 0.30 [0.18, 0.41] | 0.35 [0.22, 0.46] |

**§12 Effects on maternal body weight at the start of pregnancy**

Model 3 recovered well-established effects of maternal age, group size, and dominance status on maternal body weight at the start of pregnancy, whereby old mothers, mothers in large groups, and subordinate mothers all have lower body mass at the start of pregnancy (Figure S7).


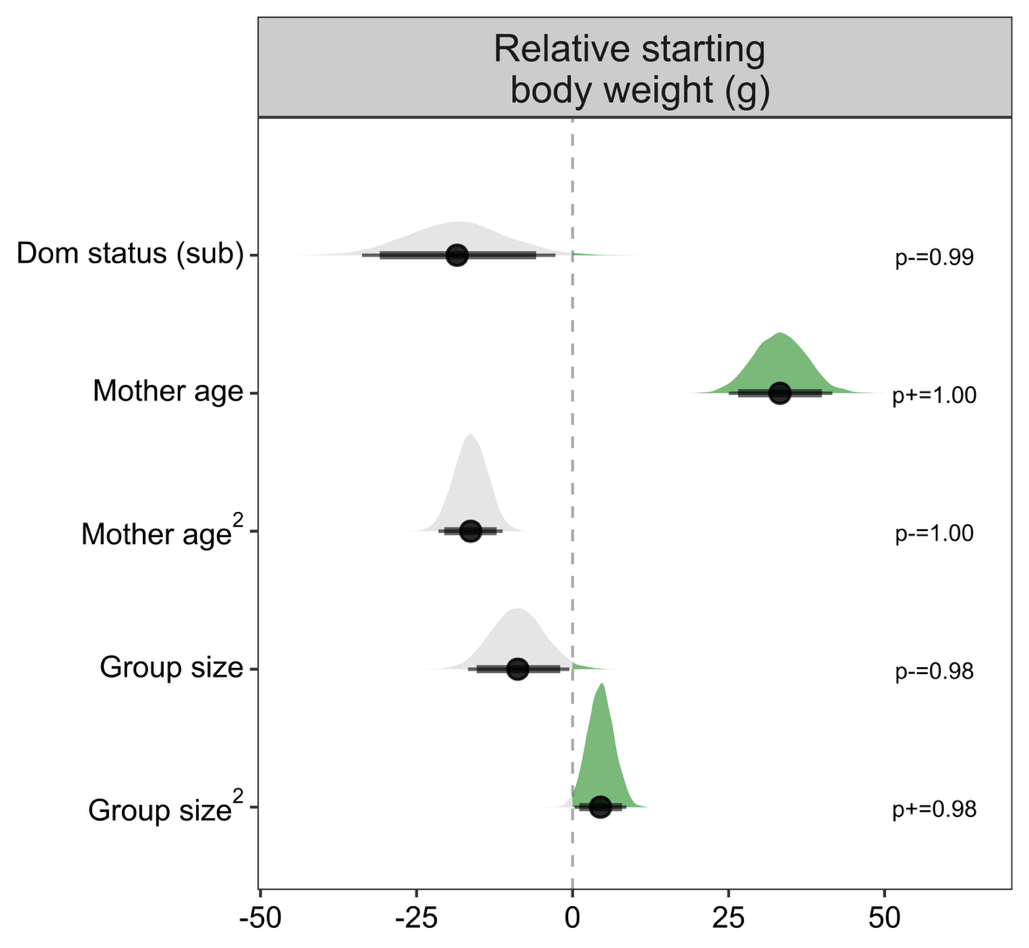


**Figure S8. The effect of dominance status, maternal age, and group size on variation in meerkat body weight at the start of pregnancy. The estimates display the relative effect of the covariate on the starting body weight and come from a model (Model 3) in which covariates were also fitted to the timing of body weight gain, and the rate of gestational weight gain (presented in main text). The full posterior distribution for each model estimate is presented, with point intervals providing the mean, 89% and 95% credible intervals. All continuous predictors were z-score transformed prior to model fitting. The probability of positive (p^+^) or negative effect (p_-_) is indicated to the right of each panel.**

**§13 Effects on the duration and rate of gestational weight gain**


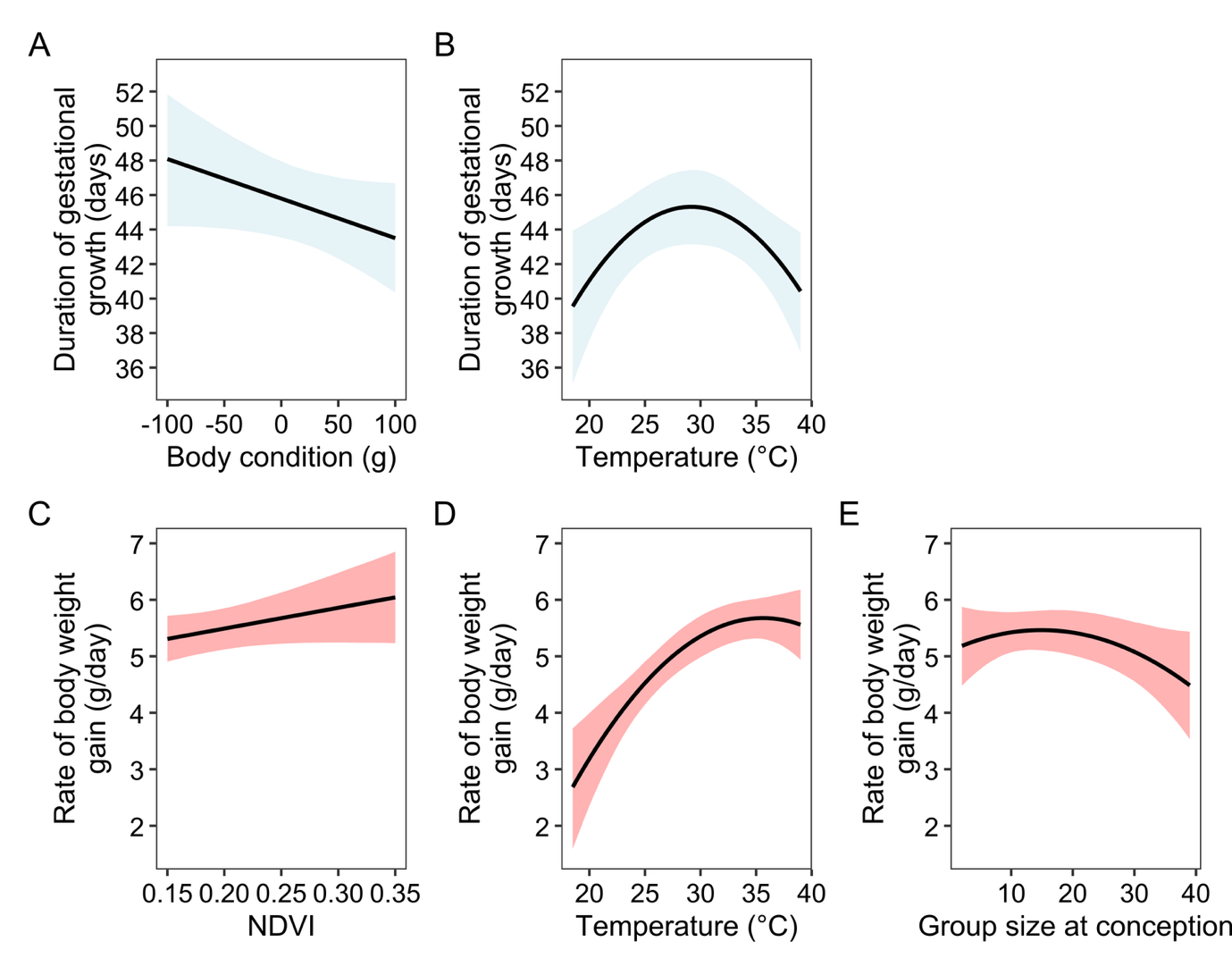


**Figure S9. Effects of various predictors on the duration and rate of gestational weight gain in wild meerkats. Figures display the predicted effect of body condition (at the start of pregnancy) on growth duration (A), average maximum daily temperature in the first half of pregnancy on growth duration (B), primary productivity (at the start of pregnancy) on gestational growth rate (C), mean maximum daily temperature on gestational growth rate (D), and group size on gestational growth rate (E). Predictions are made for dominant individuals with all other continuous variables held at their mean. Solid lines display the predicted mean response of dominant females when all other model covariates were held at their mean, with shading highlighting the 95% credible intervals conditional on population-level ‘fixed’ effects.**

**§14 Limited consequences of gestational weight gain duration on fitness**


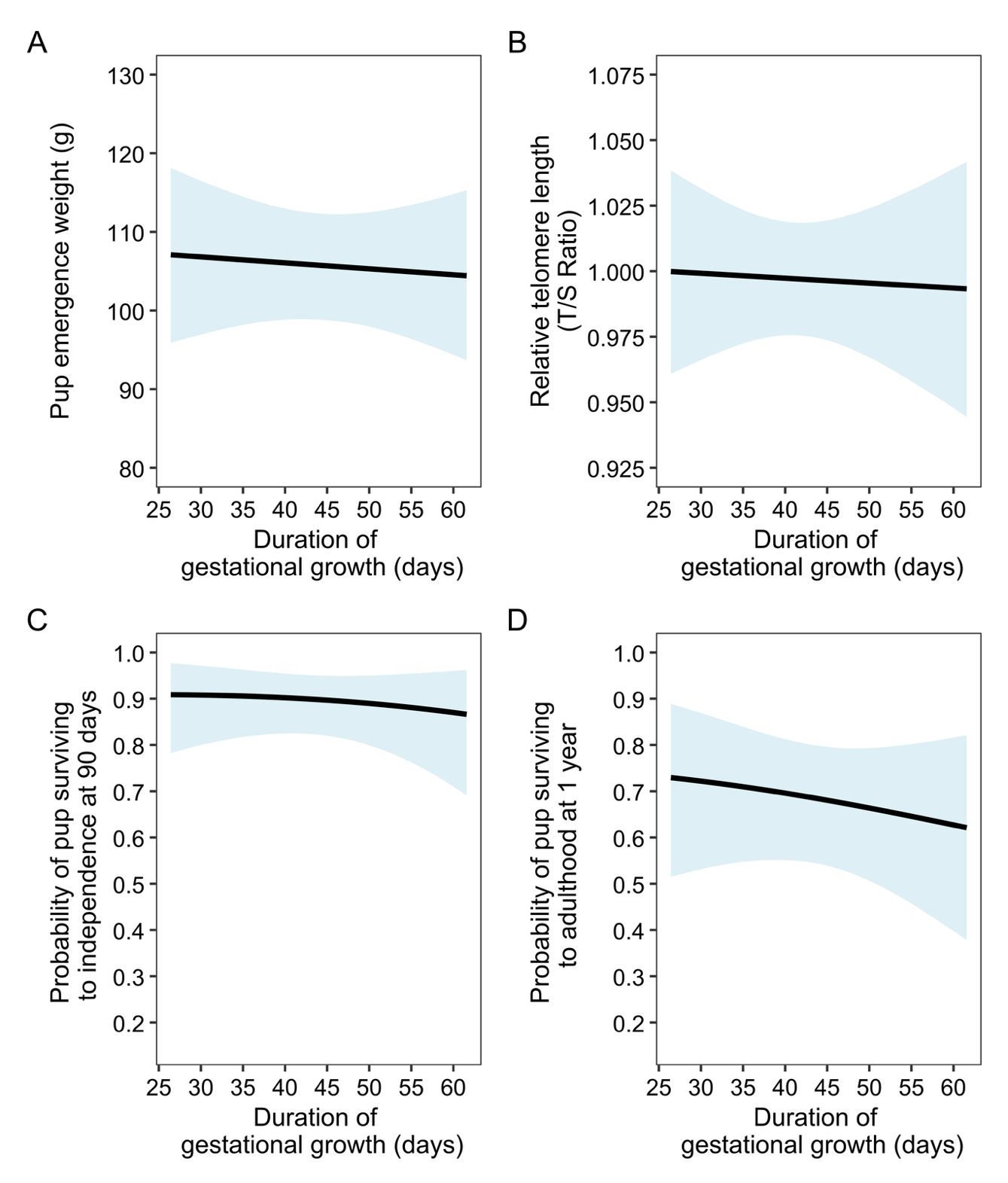


**Figure S10. The duration of gestational weight gain was not associated with any fitness measure in wild meerkats. Figures display the predicted effects of the estimated duration of growth on the weight of the pups at emergence (A) pup telomere length (B), pup survival to independence (C), and pup survival to adulthood (D, H). Solid lines display the predicted mean response of dominant females when all other model covariates were held at their mean, with shading highlighting the 95% credible intervals conditional on population-level ‘fixed’ effects.**

**§15 Supplementary Tables: Model structures and outputs**

**Table S2. Models of gestational weight dynamics fitted in the study.** All models were parameterized as a piecewise linear mixed effects model so that in addition to estimating population-level changes in the growth trajectory, we could also allow for pregnancy-specific variation in the onset and rate of gestational weight gain through random effects. Models 1, 2 & 5 also estimated the within-pregnancy correlation in shape parameters.

| **Model** | **Model purpose** | **Covariate effects on shape parameters** | **Random effects** | **Data** |
| --- | --- | --- | --- | --- |
| **1** | Estimate the raw correlation between the shape parameters of the piecewise model after controlling for litter size | *Inflection point*: litter size  *Post-inflection slope*: litter size | *Initial body weight*: Pregnancy ID  *Pre-inflection slope:* Pregnancy ID  *Inflection point:* Pregnancy ID  *Post-inflection slope:* Pregnancy ID | All pregnancies  (n = 381) |
| **2** | Estimate the raw correlation between the shape parameters of the piecewise model after controlling for litter size and other random effects | *Inflection point*: litter size  *Post-inflection slope*: litter size | *Initial body weight*: Pregnancy ID, Female ID, Breeding season  *Pre-inflection slope*: Pregnancy ID, Female ID, Breeding season  *Inflection point*: Pregnancy ID, Female ID, Breeding season  *Post-inflection slope*: Pregnancy ID, Female ID, Breeding season | All pregnancies  (n = 381) |
| **3** | Investigate the factors affecting the timing and rate of gestational growth across all pregnancies in the data set. | *Initial body weight*: Dominance status + age + age^2^ + group size + groupsize^2^  *Inflection point*: litter size + dominance status + age + age^2^ + body condition + group size + groupsize^2^ + no. other pregnant females + NDVI + temperature + temperature^2^ + dominance status:litter size + dominance status: no. other pregnant females  *Post-inflection slope*: litter size + dominance status + age + age^2^ + body condition + group size + groupsize^2^ + no. other pregnant females + NDVI + temperature + temperature^2^ + dominance status:litter size + dominance status: no. other pregnant females | *Initial body weight*: Pregnancy ID, Female ID, Breeding season  *Pre-inflection slope*: Pregnancy ID, Female ID, Breeding season  *Inflection point*: Pregnancy ID, Female ID, Breeding season  *Post-inflection slope*: Pregnancy ID, Female ID, Breeding season | All pregnancies  (n = 355) |
| **4** | Determine whether supplementary feeding affected gestational weight changes (implicating a role for maternal nutrition through pregnancy) | *Inflection point*: Litter size + treatment  *Post-inflection slope*: litter size + treatment | *Initial body weight*: Pregnancy ID  *Pre-inflection slope:* Pregnancy ID  Inflection point*:* Pregnancy ID  *Post-inflection slope:* Pregnancy ID | Supplementary feeding experiment  (n = 18: 9 fed vs 9 control) |
| **5** | Investigate whether increases in gestation length were achieved by lengthening the period from conception to the onset of gestational weight gain, the duration of gestational body weight gain, or both. | *Inflection point*: litter size + pregnancy length  *Post-inflection slope*: litter size + pregnancy length | *Initial body weight*: Pregnancy ID  *Pre-inflection slope:* Pregnancy ID  *Inflection point:* Pregnancy ID  *Post-inflection slope:* Pregnancy ID | Pregnancies with a recorded estrus/mating event at the start  (n = 76) |

**Table S3. Model 1 output.** Estimates present the posterior mean and its standard deviation, alongside the associated 95% credible intervals (CrI) for the lower and upper bound. Residual error [std dev] = 18.58 ± 0.13 (95% CrI: 18.33, 18.84). Meerkat litter size was z-score transformed prior to model fitting (mean = 3.57, 1SD = 1.37). p_+/-_ is the ‘probability of direction’: the proportion of the posterior distribution that is greater than 0 in the direction of the median.

| **Population-level “fixed” effects** | **Mean (SD)** | **Lower 95% CrI** | **Upper 95%  CrI** | **p_+/-_** |
| --- | --- | --- | --- | --- |
| *Shape parameters* |  |  |  |  |
| Initial body weight ($\beta_{10}$) [*grams*] | 743.50 (4.38) | 735.04 | 752.09 |  |
| Pre-inflection slope ($\beta_{20}$) [*g/day*] | -0.26 (0.11) | -0.48 | -0.04 | - 0.99 |
| Inflection point ($\omega_{0}$) [*days*] | 30.83 (0.49) | 29.87 | 31.79 |  |
| Post-inflection slope ($\beta_{30})$[*g/day*] | 5.20 (0.08) | 5.04 | 5.37 | + 1.00 |
|  |  |  |  |  |
| *Inflection point* *~* |  |  |  |  |
| Litter size | 0.30 (0.48) | -0.65 | 1.24 | + 0.73 |
|  |  |  |  |  |
| *Post-inflection slope ~* |  |  |  |  |
| Litter size | 0.66 (0.09) | 0.49 | 0.83 | + 1.00 |
|  |  |  |  |  |
| **Group-level “random” effects** | **Mean (SD)** | **Lower 95% CrI** | **Upper 95%  CrI** | **p_+/-_** |
| **Pregnancy ID (n = 381)** |  |  |  |  |
| std dev. Initial body weight | 85.52 (3.25) | 79.33 | 92.02 |  |
| std dev. Pre-inflection slope | 2.00 (0.08) | 1.85 | 2.18 |  |
| std dev. Inflection point | 7.75 (0.41) | 6.99 | 8.58 |  |
| std dev. Post-inflection slope | 1.59 (0.06) | 1.47 | 1.72 |  |
|  |  |  |  |  |
| *corr* Initial body weight - Inflection point | 0.10 (0.06) | -0.02 | 0.22 | + 0.95 |
| *corr* Initial body weight - Post-inflection slope | 0.04 (0.06) | -0.06 | 0.15 | + 0.78 |
| *corr* Inflection point - Post-inflection slope | 0.30 (0.06) | 0.18 | 0.41 | + 1.00 |

**Table S4. Model 2 output.** Estimates present the posterior mean and its standard deviation, alongside the associated 95% credible intervals (CrI) for the lower and upper bound. Residual error [std dev] = 18.59 ± 0.13 (95% CrI: 18.35, 18.85). Meerkat litter size was z-score transformed prior to model fitting (mean = 3.57, 1SD = 1.37). Breeding season refers to yearly periods from July to June. p_+/-_ is the ‘probability of direction’: the proportion of the posterior distribution that is greater than 0 in the direction of the median.

| **Population-level “fixed” effects** | **Mean (SD)** | **Lower 95%  CrI** | **Upper 95% CrI** | **p_+/-_** |
| --- | --- | --- | --- | --- |
| *Shape parameters* |  |  |  |  |
| Initial body weight ($\beta_{10}$) [*grams*] | 711.44 (8.24) | 694.94 | 727.37 |  |
| Pre-inflection slope ($\beta_{20}$) [*g/day*] | -0.22 (0.13) | -0.48 | 0.04 | - 0.95 |
| Inflection point ($\omega_{0}$) [*days*] | 30.84 (0.74) | 29.40 | 32.31 |  |
| Post-inflection slope ($\beta_{30})$ [*g/day*] | 5.16 (0.12) | 4.93 | 5.39 | + 1.00 |
|  |  |  |  |  |
| *Inflection point* *~* |  |  |  |  |
| Litter size | 0.50 (0.47) | -0.43 | 1.43 | + 0.86 |
|  |  |  |  |  |
| *Post-inflection slope ~* |  |  |  |  |
| Litter size | 0.68 (0.09) | 0.51 | 0.84 | + 1.00 |
|  |  |  |  |  |
| **Group-level “random” effects** | **Mean (SD)** | **Lower 95%  CrI** | **Upper 95% CrI** | **p_+/-_** |
| **Pregnancy ID (n = 381)** |  |  |  |  |
| std dev. Initial body weight | 46.17 (2.47) | 41.55 | 51.26 |  |
| std dev. Pre-inflection slope | 1.95 (0.09) | 1.78 | 2.13 |  |
| std dev. Inflection point | 7.38 (0.43) | 6.56 | 8.25 |  |
| std dev. Post-inflection slope | 1.53 (0.07) | 1.40 | 1.66 |  |
|  |  |  |  |  |
| *corr* Initial body weight - Inflection point | 0.07 (0.08) | -0.08 | 0.21 | + 0.82 |
| *corr* Initial body weight - Post-inflection slope | -0.06 (0.07) | -0.19 | 0.07 | - 0.82 |
| *corr* Inflection point - Post-inflection slope | 0.35 (0.06) | 0.22 | 0.46 | + 1.00 |
|  |  |  |  |  |
| **Female ID (n = 135)** |  |  |  |  |
| std dev. Initial body weight | 75.13 (5.94) | 64.17 | 87.48 |  |
| std dev. Pre-inflection slope | 0.35 (0.20) | 0.02 | 0.74 |  |
| std dev. Inflection point | 1.35 (0.87) | 0.07 | 3.21 |  |
| std dev. Post-inflection slope | 0.43 (0.14) | 0.13 | 0.70 |  |
|  |  |  |  |  |
| **Breeding season (n = 24)** |  |  |  |  |
| std dev. Initial body weight | 17.13 (5.48) | 6.48 | 28.31 |  |
| std dev. Pre-inflection slope | 0.23 (0.16) | 0.01 | 0.58 |  |
| std dev. Inflection point | 2.30 (0.93) | 0.52 | 4.19 |  |
| std dev. Post-inflection slope | 0.20 (0.13) | 0.01 | 0.50 |  |

**Table S5. Model 3 output: maternal, social, and environmental effects on variation in gestational body weight in wild pregnant meerkats.** The model was parameterized so that the various predictors could influence the shape parameters independently. Estimates present the posterior mean and its standard deviation, alongside the associated 95% credible intervals (CrI) for the lower and upper bound. All continuous predictors were z-score transformed prior to model fitting. This means that for model covariates with both linear and quadratic components, the linear component indicates the rate of change when the covariate is at the mean value of the covariate, while the quadratic component indicates the direction and steepness of the curvature at this point. Residual error [std dev] = 18.71 ± 0.13 (95% CrI: 18.45, 18.98). p_+/-_ is the ‘probability of direction’: the proportion of the posterior distribution that is greater than 0 in the direction of the median. For the interactions we also include the probability of direction for the total effect in square bracket, which indicates the probability that the slope for the effects of litter size or the number of pregnant females is positive or negative in subordinates.

| **Population-level “fixed” effects** | **Mean (SD)** | **Lower 95% CrI** | **Upper 95%  CrI** | **p_+/-_** |
| --- | --- | --- | --- | --- |
| *Shape parameters* |  |  |  |  |
| Initial body weight ($\beta_{1i}$) [*grams*] | 747.73 (8.37) | 731.08 | 763.80 |  |
| Pre-inflection slope ($\beta_{2i}$ ) [*g/day*] | -0.13 (0.14) | -0.40 | 0.15 | - 0.83 |
| Inflection point ($\omega_{i}$) [*days*] | 29.70 (1.09) | 27.56 | 31.86 |  |
| Post-inflection slope ($\beta_{3i})$ [*g/day*] | 5.46 (0.18) | 5.09 | 5.82 | + 1.00 |
|  |  |  |  |  |
| *Initial body weight ~* |  |  |  |  |
| Dominant status: subordinate | -18.51 (7.86) | -33.78 | -2.76 | - 0.99 |
| Mother age | 32.24 (4.23) | 25.04 | 41.63 | + 1.00 |
| Mother age^2^ | -16.32 (2.64) | -21.48 | -11.20 | - 1.00 |
| Group size | -8.78 (4.20) | -16.76 | -0.52 | - 0.98 |
| Group size^2^ | 4.48 (2.13) | 0.32 | 8.64 | + 0.98 |
|  |  |  |  |  |
| *Inflection point* *~* |  |  |  |  |
| Litter size | 0.74 (0.54) | -0.33 | 1.78 | + 0.91 |
| Dom status (sub) | 0.79 (1.90) | -2.91 | 4.55 | + 0.66 |
| Mother age | -0.03 (0.73) | -1.46 | 1.41 | + 0.51 |
| Mother age^2^ | 0.44 (0.43) | -0.41 | 1.28 | + 0.85 |
| Body condition | 0.92 (0.57) | -0.19 | 2.04 | + 0.95 |
| Group size | 0.33 (0.67) | -0.99 | 1.64 | + 0.69 |
| Group size^2^ | -0.28 (0.41) | -1.10 | 0.52 | - 0.75 |
| No. other pregnant females | -0.17 (0.56) | -1.26 | 0.92 | - 0.62 |
| Temperature | 0.28 (0.56) | -0.84 | 1.37 | + 0.69 |
| Temperature^2^ | 1.53 (0.59) | 0.38 | 2.69 | + 1.00 |
| NDVI | -0.02 (0.51) | -1.02 | 0.97 | - 0.51 |
| Dom status (sub):Litter size | -1.78 (1.48) | -4.69 | 1.11 | - 0.88 [- 0.77] |
| Dom status (sub):No. other preg fems | -1.22 (1.05) | -3.27 | 0.80 | -0.87 [- 0.94] |
|  |  |  |  |  |
| *Post-inflection slope ~* |  |  |  |  |
| Litter size | 0.64 (0.09) | 0.46 | 0.83 | + 1.00 |
| Dominant status(sub) | -0.46 (0.34) | -1.13 | 0.21 | - 0.91 |
| Mother age | 0.06 (0.12) | -0.18 | 0.30 | + 0.68 |
| Mother age^2^ | 0.04 (0.08) | -0.11 | 0.19 | + 0.70 |
| Body condition | -0.00 (0.09) | -0.17 | 0.17 | - 0.50 |
| Group size | -0.03 (0.12) | -0.26 | 0.20 | - 0.61 |
| Group size^2^ | -0.09 (0.06) | -0.21 | 0.02 | - 0.94 |
| No. other pregnant females | -0.01 (0.10) | -0.20 | 0.17 | + 0.56 |
| Temperature | 0.45 (0.10) | 0.26 | 0.64 | + 1.00 |
| Temperature^2^ | -0.23 (0.09) | -0.42 | -0.05 | - 0.99 |
| NDVI | 0.14 (0.09) | -0.03 | 0.30 | + 0.94 |
| Dom status (sub):Litter size | -0.67 (0.25) | -1.17 | -0.20 | - 1.00 [ -0.54] |
| Dom status (sub):No. other preg fems | 0.08 (0.18) | -0.43 | 0.27 | + 0.67 [- 0.72] |

|  |  |  |  |  |
| --- | --- | --- | --- | --- |
| **Group-level “random” effects** | **Mean (SD)** | **Lower 95% CrI** | **Upper 95% CrI** |  |
| **Pregnancy ID (n = 355)** |  |  |  |  |
| std dev. Initial body weight | 41.87 (2.34) | 37.54 | 46.68 |  |
| std dev. Pre-inflection slope | 1.93 (0.09) | 1.76 | 2.12 |  |
| std dev. Inflection point | 7.34 (0.44) | 6.49 | 8.22 |  |
| std dev. Post-inflection slope | 1.34 (0.07) | 1.21 | 1.47 |  |
|  |  |  |  |  |
| **Female ID (n = 130)** |  |  |  |  |
| std dev. Initial body weight | 57.61 (5.31) | 47.91 | 68.65 |  |
| std dev. Pre-inflection slope | 0.42 (0.21) | 0.04 | 0.83 |  |
| std dev. Inflection point | 1.40 (0.95) | 0.06 | 3.52 |  |
| std dev. Post-inflection slope | 0.60 (0.13) | 0.34 | 0.85 |  |
|  |  |  |  |  |
| **Breeding season (n = 22)** |  |  |  |  |
| std dev. Initial body weight | 15.60 (5.99) | 3.15 | 27.67 |  |
| std dev. Pre-inflection slope | 0.20 (0.15) | 0.01 | 0.56 |  |
| std dev. Inflection point | 1.47 (0.89) | 0.09 | 3.38 |  |
| std dev. Post-inflection slope | 0.17 (0.12) | 0.01 | 0.44 |  |

**Table S6. Model 4 output: The effect of supplemental feeding throughout pregnancy on gestational body weight in wild meerkats.** The model was parameterized so that the effect of treatment (Fed vs Control) could influence the shape parameters independently. Estimates present the posterior mean and its standard deviation, alongside the associated 95% credible intervals (CrI) for the lower and upper bound. All predictors were z-score transformed prior to model fitting. Residual error [std dev] = 18.37 ± 0.55 (95% CrI: 17.33, 19.50). p_+/-_ is the ‘probability of direction’: the proportion of the posterior distribution that is greater than 0 in the direction of the median.

| **Population-level “fixed” effects** | **Mean (SD)** | **Lower 95%  CrI** | **Upper 95% CrI** | **p_+/-_** |
| --- | --- | --- | --- | --- |
| *Shape parameters* |  |  |  |  |
| Initial body weight ($\beta_{10}$) [*grams*] | 751.75 (20.70) | 710.53 | 792.88 |  |
| Pre-inflection slope ($\beta_{20}$ ) [*g/day*] | -0.65 (0.69) | -2.01 | 0.73 | - 0.83 |
| Inflection point ($\omega_{0}$) [*days*] | 31.57 (2.77) | 25.93 | 36.95 |  |
| Post-inflection slope ($\beta_{30})$ [*g/day*] | 4.63 (0.71) | 3.25 | 6.08 | + 1.00 |
|  |  |  |  |  |
| *Inflection* *point* *~* |  |  |  |  |
| Treatment: Fed | 1.27 (3.15) | -4.93 | 7.49 | + 0.66 |
| Litter size | -1.06 (1.92) | -4.89 | 2.79 | - 0.72 |
|  |  |  |  |  |
| *Post-inflection slope ~* |  |  |  |  |
| Treatment: Fed | 2.12 (1.03) | 0.04 | 4.12 | + 0.98 |
| Litter size | 0.69 (0.55) | -0.39 | 1.77 | +0.91 |
|  |  |  |  |  |
| **Group-level “random” effects** | **Mean (SD)** | **Lower 95%  CrI** | **Upper 95% CrI** |  |
| **Pregnancy ID (n = 18)** |  |  |  |  |
| std dev. Initial body weight | 89.95 (15.36) | 65.87 | 125.06 |  |
| std dev. Pre-inflection slope | 2.88 (0.53) | 2.04 | 4.12 |  |
| std dev. Inflection point | 7.50 (1.88) | 4.70 | 11.91 |  |
| std dev. Post-inflection slope | 2.21 (0.42) | 1.54 | 3.18 |  |

**Table S7. Model output: factors affecting gestation length in wild meerkats.** The linear model was fit to 70 pregnancies that had a known start date tied to observations of mating or oestrus. Estimates present the posterior mean and its standard deviation, alongside the associated 95% credible intervals (CrI) for the lower and upper bound. All predictors were z-score transformed prior to model fitting. Residual error [std dev] = 2.62 ± 0.26 (95% CrI: 2.16, 3.18). p_+/-_ is the ‘probability of direction’: the proportion of the posterior distribution that is greater than 0 in the direction of the median.

| **Population-level “fixed” effects** | **Mean (SD)** | **Lower 95%  CrI** | **Upper 95% CrI** | **p_+/-_** |
| --- | --- | --- | --- | --- |
| Intercept | 73.87 (0.53) | 72.84 | 74.91 |  |
| Litter size | -0.30 (0.36) | -1.02 | 0.41 | - 0.80 |
| Body condition | -0.68 (0.39) | -1.45 | 0.09 | - 0.96 |
| Group size | -1.08 (0.36) | -1.81 | -0.37 | - 1.00 |
| No. other preg fems | 0.32 (0.34) | -0.34 | 0.98 | + 0.83 |
| NDVI | 0.64 (0.36) | -0.06 | 1.34 | + 0.96 |
| Temperature | -1.01 (0.46) | -1.94 | -0.12 | - 0.99 |
| Temperature^2^ | 0.69 (0.39) | -0.07 | 1.45 | + 0.96 |
|  |  |  |  |  |
| **Group-level “random” effects** | **Mean (SD)** | **Lower 95%  CrI** | **Upper 95% CrI** |  |
| Female ID std dev. (n = 37) | 0.70 (0.48) | 0.03 | 1.77 |  |


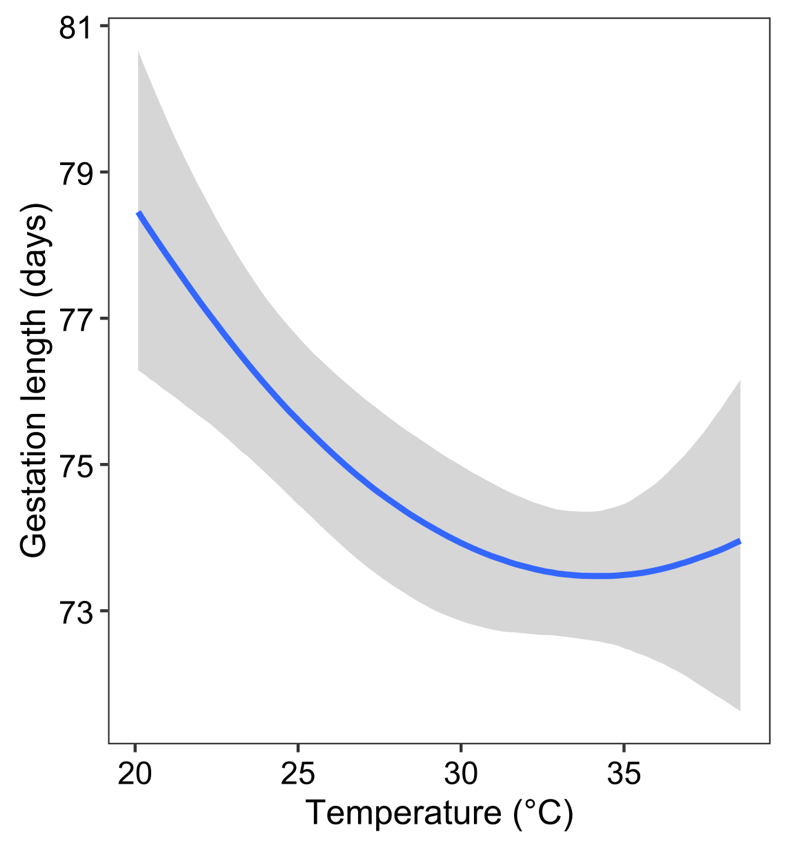


**Figure S11. The quadratic effect of temperature on gestation lengths in wild meerkats. Increases in temperature are associated with shorter gestation lengths, up until around 30ºC, after which point the average gestation length levels off. The plot shows the mean effect of temperature (± 95% credible interval), conditional of all other terms in the model being held at their mean level.**

**Table S8. Model 5 output: body weight changes for meerkat pregnancies that started with an observed oestrus or mating event.** The purpose of the model was to determine whether increases in pregnancy length were achieved through a lengthening in the period from conception to the onset of weight gain, from the onset of weight gain to birth, or both. Estimates present the posterior mean and its standard deviation, alongside the associated 95% credible intervals (CrI) for the lower and upper bound. Residual error [std dev] = 20.07 ± 0.30 (95% CrI: 19.50, 20.66). p_+/-_ is the ‘probability of direction’: the proportion of the posterior distribution that is greater than 0 in the direction of the median. While litter size was z-score transformed for model fitting, the pregnancy length term was kept on its raw scale. Consequently, the parameter estimate for this term indicates that for every 1-day increase in pregnancy length, the inflection gets relatively earlier, implying that longer pregnancies are achieved through proportionally greater extensions to pre-inflection period than the post-inflection period (the duration of growth).

| **Population-level “fixed” effects** | **Mean (SD)** | **Lower 95%  CrI** | **Upper 95% CrI** | **p_+/-_** |
| --- | --- | --- | --- | --- |
| *Shape parameters* |  |  |  |  |
| Initial body weight ($\beta_{10}$) [*grams*] | 754.50 (12.05) | 730.47 | 777.88 |  |
| Pre-inflection slope ($\beta_{20}$) [*g/day*] | -0.46 (0.29) | -1.03 | 0.11 | - 0.95 |
| Inflection point ($\omega_{0}$) [*days*] | 29.69 (1.13) | 27.47 | 31.90 |  |
| Post-inflection slope ($\beta_{30})$ [*g/day*] | 5.52 (0.20) | 5.13 | 5.92 | + 1.00 |
|  |  |  |  |  |
| *Inflection point* *~* |  |  |  |  |
| Litter size | 1.77 (0.98) | -0.15 | 3.72 | + 0.96 |
| Pregnancy length | 0.73 (0.33) | 0.08 | 1.38 | + 0.98 |
|  |  |  |  |  |
| *Post-inflection slope ~* |  |  |  |  |
| Litter size | 1.31 (0.18) | 0.96 | 1.67 | + 1.00 |
| Pregnancy length | -0.10 (0.60) | -0.22 | 0.02 | - 0.95 |
|  |  |  |  |  |
| **Group-level “random” effects** | **Mean (SD)** | **Lower 95%  CrI** | **Upper 95% CrI** | **p_+/-_** |
| **Pregnancy ID (n = 76)** |  |  |  |  |
| std dev. Initial body weight | 40.96 (5.78) | 21.52 | 53.83 |  |
| std dev. Pre-inflection slope | 2.01 (0.24) | 1.56 | 2.49 |  |
| std dev. Inflection point | 6.81 (0.89) | 5.20 | 8.68 |  |
| std dev. Post-inflection slope | 0.55 (0.29) | 0.04 | 1.13 |  |
|  |  |  |  |  |
| *corr* Initial body weight - Inflection point | 0.13 (0.18) | -0.22 | 0.47 | + 0.77 |
| *corr* Initial body weight - Post-inflection slope | 0.07 (0.16) | -0.25 | 0.37 | + 0.68 |
| *corr* Inflection point - Post-inflection slope | 0.31 (0.14) | 0.01 | 0.57 | + 0.98 |
|  |  |  |  |  |
| **Female ID (n = 40)** |  |  |  |  |
| std dev. Initial body weight | 66.33 (10.62) | 46.79 | 88.47 |  |
| std dev. Pre-inflection slope | 0.73 (0.43) | 0.04 | 1.63 |  |
| std dev. Inflection point | 2.31 (1.57) | 0.10 | 5.84 |  |
| std dev. Post-inflection slope | 0.55 (0.29) | 0.04 | 1.13 |  |

**Table S9. The effect of gestational weight gain parameters on the body weight of meerkat pups at emergence from the birth burrow (approximately 18 days).** The model table displays the combined posterior distribution of 100 sub-models (Gaussian linear mixed effects models). Each sub-model was fitted to a data set containing unique estimates for the duration of gestational weight gain (‘growth duration’) and the post-inflection slope for each pregnancy - taken from the posterior distributions of Model 1 (see main text). All other covariates were consistent across sub-models. Estimates present the posterior mean and its standard deviation, alongside the associated 95% credible intervals (CrI) for the lower and upper bound. All continuous predictors were z-score transformed prior to model fitting. Data was fitted to 971 pups that emerged from 289 litters (for which there was weights information). Residual error [std dev] = 8.75 ± 0.24 (95% CrI: 8.30, 9.23). p_+/-_ is the ‘probability of direction’: the proportion of the posterior distribution that is greater than 0 in the direction of the median.

| **Population-level “fixed” effects** | **Mean (SD)** | **Lower 95% CrI** | **Upper 95%  CrI** | **p_+/-_** |
| --- | --- | --- | --- | --- |
| Intercept | 105.76 (3.43) | 98.84 | 112.35 |  |
| Growth duration estimate | -0.53 (1.77) | -4.00 | 2.95 | - 0.62 |
| *Per capita* post-inflection slope estimate | 3.55 (1.82) | -0.01 | 7.13 | + 0.97 |
| Age of first weight | 7.65 (0.86) | 5.97 | 9.32 | + 1.00 |
| Mother status: subordinate | 7.89 (5.72) | -3.33 | 19.18 | + 0.92 |
| Mother age | -1.72 (2.30) | -6.25 | 2.78 | - 0.77 |
| Mother age^2^ | -3.11 (1.35) | -5.76 | -0.47 | - 0.99 |
| Group size at birth | -3.81 (1.91) | -7.59 | -0.08 | - 0.98 |
|  |  |  |  |  |
| **Group-level “random” effects** | **Mean (SD)** | **Lower 95% CrI** | **Upper 95%  CrI** |  |
| Pregnancy ID (n = 289) | 21.70 (1.31) | 19.28 | 24.42 |  |
| Female ID (n = 118) | 13.00 (2.67) | 7.63 | 18.18 |  |
| Breeding season (n = 24) | 10.06 (3.26) | 4.03 | 16.99 |  |

**Table S10. The effect of gestational weight gain parameters on meerkat pup telomere length at emergence.** The model table displays the combined posterior distribution of 100 sub-models (Gaussian linear mixed effects models). Each sub-model was fitted to a data set containing unique estimates for the duration of gestational weight gain (‘growth duration’) and the post-inflection slope for each pregnancy- taken from the posterior distributions of Model 1 (see main text). All other covariates were consistent across models. Estimates present the posterior mean and its standard deviation, alongside the associated 95% credible intervals (CrI) for the lower and upper bound. All continuous predictors were z-score transformed prior to model fitting, and all estimates are presented on the logit-scale. Data was fitted to 159 pups that emerged from 42 litters). Residual error [std dev] = 0.05 ± 0.003 (95% CrI: 0.05, 0.06). p_+/-_ is the ‘probability of direction’: the proportion of the posterior distribution that is greater than 0 in the direction of the median.

| **Population-level “fixed” effects** | **Mean (SD)** | **Lower 95% CrI** | **Upper 95%  CrI** | **p_+/-_** |
| --- | --- | --- | --- | --- |
| Intercept | 1.00 (0.01) | 0.98 | 1.02 |  |
| Growth duration estimate | -0.002 (0.01) | -0.02 | 0.01 | - 0.62 |
| *Per capita* post-inflection slope estimate | -0.009 (0.02) | -0.04 | 0.02 | - 0.72 |
| Age at sampling/capture | 0.006 (0.01) | -0.01 | 0.02 | + 0.86 |
| Mother status: subordinate | 0.004 (0.04) | -0.07 | 0.08 | + 0.54 |
| Mother age | 0.02 (0.01) | -0.01 | 0.04 | + 0.89 |
| Group size at birth | -0.003 (0.01) | -0.02 | 0.01 | - 0.65 |
|  |  |  |  |  |
| **Group-level “random” effects** | **Mean (SD)** | **Lower 95% CrI** | **Upper 95%  CrI** |  |
| Pregnancy ID (n = 42) | 0.02 (0.01) | 0.003 | 0.04 |  |
| Female ID (n = 18) | 0.013 (0.01) | 0.001 | 0.03 |  |

**Table S11. The effect of gestational weight gain parameters on meerkat pup survival to independence (90 days).** The model table displays the combined posterior distribution of 100 sub-models (Bernoulli linear mixed effects models). Each sub-model was fitted to a data set containing unique estimates for the duration of gestational weight gain (‘growth duration’) and the post-inflection slope for each pregnancy - taken from the posterior distributions of Model 1 (see main text). All other covariates were consistent across models. Estimates present the posterior mean and its standard deviation, alongside the associated 95% credible intervals (CrI) for the lower and upper bound, on the logit scale. All continuous predictors were z-score transformed prior to model fitting, and all estimates are presented on the logit-scale. Data was fitted to 960 pups that emerged from 287 litters. p_+/-_ is the ‘probability of direction’: the proportion of the posterior distribution that is greater than 0 in the direction of the median.

| **Population-level “fixed” effects** | **Mean (SD)** | **Lower 95% CrI** | **Upper 95% CrI** | **p_+/-_** |
| --- | --- | --- | --- | --- |
| Intercept | 2.23 (0.36) | 1.53 | 2.95 |  |
| Growth duration estimate | -0.90 (0.20) | -0.50 | 0.30 | - 0.67 |
| *Per capita* post-inflection slope estimate | -0.23 (0.23) | -0.67 | 0.22 | - 0.85 |
| Emergence weight* | 1.06 (0.20) | 0.69 | 1.46 | + 1.00 |
| Mother status: subordinate | 0.003 (0.63) | -1.22 | 1.25 | + 0.50 |
| Mother age | 0.12 (0.24) | -0.35 | 0.60 | + 0.69 |
| Mother age^2^ | -0.30 (0.15) | -0.59 | -0.02 | - 0.98 |
| Group size at birth | 0.33 (0.22) | -0.09 | 0.76 | + 0.94 |
|  |  |  |  |  |
| **Group-level “random” effects** | **Mean (SD)** | **Lower 95% CrI** | **Upper 95% CrI** |  |
| Pregnancy ID (n = 287) | 1.65 (0.30) | 1.07 | 2.26 |  |
| Female ID (n = 117) | 0.82 (0.41) | 0.06 | 1.61 |  |
| Breeding season (n = 24) | 0.86 (0.35) | 0.20 | 1.62 |  |

*The emergence weight was standardized to 25 days of age for all pups in the data set (as described in the Supplementary Material above). Refitting the model to instead use the measured emergence weight for each pup - which did not correct for their age – produced an estimate for the emergence term of 1.08 ± 0.19 (95% CrI: 0.72, 1.48; p_+_= 1.00). The estimate for the *per capita* post-inflection slope in this model became -0.08 ± 0.20 (95% CrI: -0.48, 0.31; p_-_= 0.78). Thus, increases in the rate of gestational weight gain [per pup] did not directly affect pup survival to independence, but did affect survival indirectly by positively impacting pup body weight at emergence (which itself increased survival: Table S9).

**Table S12. Model output: the effect of gestational weight gain parameters on meerkat pup survival to adulthood (1 year).** The model table displays the combined posterior distribution of 100 sub-models (Bernoulli linear mixed effects models). Each sub-model was fitted to a data set containing unique estimates for the duration of gestational weight gain (‘growth duration’) and the post- inflection slope for each pregnancy taken from the posterior distributions of Model 1 (see main text). All other covariates were consistent across models. Estimates present the posterior mean and its standard deviation, alongside the associated 95% credible intervals (CrI) for the lower and upper bound, on the logit scale. All continuous predictors were z-score transformed prior to model fitting, and all estimates are presented on the logit-scale. Data was fitted to 945 pups that emerged from 283 litters. p_+/-_ is the ‘probability of direction’: the proportion of the posterior distribution that is greater than 0 in the direction of the median.

| **Population-level “fixed” effects** | **Mean (SD)** | **Lower 95% CrI** | **Upper 95% CrI** | **p_+/-_** |
| --- | --- | --- | --- | --- |
| Intercept | 0.79 (0.30) | 0.17 | 1.38 |  |
| Growth duration estimate | -0.11 (0.17) | -0.44 | 0.21 | - 0.73 |
| *Per capita* post-inflection slope estimate | 0.06 (0.20) | -0.44 | 0.21 | + 0.61 |
| Emergence weight* | 0.67 (0.14) | 0.40 | 0.97 | + 1.00 |
| Mother Status: subordinate | 0.24 (0.50) | -0.73 | 1.24 | + 0.68 |
| Mother age | 0.11 (0.20) | -0.29 | 0.50 | + 0.71 |
| Mother age^2^ | -0.17 (0.12) | -0.41 | 0.06 | - 0.91 |
| Group size at birth | 0.27 (0.17) | -0.07 | 0.61 | + 0.94 |
|  |  |  |  |  |
| **Group-level “random” effects** | **Mean (SD)** | **Lower 95% CrI** | **Upper 95% CrI** |  |
| Pregnancy ID (n = 283) | 1.25 (0.22) | 0.81 | 1.69 |  |
| Female ID (n = 116) | 0.69 (0.35) | 0.05 | 1.38 |  |
| Breeding season (n = 24) | 1.25 (0.22) | 0.81 | 1.69 |  |

*The emergence weight was standardized to 25 days of age for all pups in the data set (as described above, §8). Refitting the model to instead use the observed emergence weight for each pup - which did not correct for their age – produced an estimate for the emergence term of 0.63 ± 0.14 (95% CrI: 0.37, 0.92; p_+_= 1.00). The estimate for the *per capita* post-inflection slope in this model became 0.10 ± 0.20 (95% CrI: -0.29, 0.49; p_+_= 0.68). Thus, increases in the rate of gestational weight gain [per pup] did not directly affect pup survival to adulthood, but did affect survival indirectly by positively impacting pup body weight at emergence (which itself increased survival).

**SUPPLEMENTARY REFERENCES**

Cawthon R.M., Smith K.R., O'Brien E., Sivatchenko A., & Kerber R.A. (2003) Association between telomere length in blood and mortality in people aged 60 years or older. *Lancet*, **361** doi:10.1016/s0140-6736(03)12384-7

Criscuolo F., Bize P., Nasir L., Metcalfe N.B., Foote C.G., Griffiths K., Gault E.A., & Monaghan P. (2009) Real-time quantitative PCR assay for measurement of avian telomeres. *Journal of Avian Biology*, **40**(3), 342-347. doi:10.1111/j.1600-048X.2008.04623.x.

Crottini A., Madsen O., Poux C., Strauß A., Vieites D.R., & Vences M. (2012) Vertebrate time-tree elucidates the biogeographic pattern of a major biotic change around the K–T boundary in Madagascar. *Proceedings of the National Academy of Sciences*, **109**(14), 5358-5363. doi:10.1073/pnas.1112487109

Dalerum, F., Bennett, N. C., & Clutton-Brock, T. H. (2007). Longitudinal differences in 15N between mothers and offspring during and after weaning in a small cooperative mammal, the meerkat (*Suricata suricatta*). *Rapid Communications in Mass Spectrometry*, *21*(12), 1889–1892. https://doi.org/10.1002/rcm.3032

Doolan, S.P., & MacDonald, D.W. (1997) Breeding and juvenile survival among slender-tailed meerkats (Suricata suricatta) in the south-western Kalahari: ecological and social influences. *Journal of Zoology*, **242**(2), 309-327. doi:10.1111/j.1469-7998.1997.tb05804.x

English S., Bateman A.W., & Clutton-Brock T.H. (2012) Lifetime growth in wild meerkats: incorporating life history and environmental factors into a standard growth model. *Oecologia*, **169**(1), 143-153. doi:10.1007/s00442-011-2192-9

Epel E.S., Blackburn E.H., Lin J., Dhabhar F.S., Adler N.E., Morrow J.D., & Cawthon R.M. (2004) Accelerated telomere shortening in response to life stress. *Proceedings of the National Academy of Sciences of the United States of America*, **101**(49), 17312-17315. doi:10.1073/pnas.0407162101

Griffin A.S., Pemberton J.M., Brotherton P.N.M., McIlrath G., Gaynor D., Kansky R., O'Riain J., & Clutton-Brock T.H. (2003) A genetic analysis of breeding success in the cooperative meerkat (*Suricata suricatta*). *Behavioral Ecology*, **14**(4), 472-480. doi:10.1093/beheco/arg040.

Hellemans J., Mortier G., De Paepe A., Speleman F., & Vandesompele J. (2007) qBase relative quantification framework and software for management and automated analysis of real-time quantitative PCR data. *Genome Biology*, **8**(2), R19. doi:10.1186/gb-2007-8-2-r19

Hufkens, K. (2022) The MODISTools package: an interface to the MODIS Land Products Subsets Web Services. https://github.com/ropensci/MODISTools

Inzani E.L., Marshall H.H., Sanderson J.L., Nichols H.J., Thompson F.J., Kalema-Zikusoka G., Hodge S.J., Cant M.A., & Vitikainen E.I.K. (2016) Female reproductive competition explains variation in prenatal investment in wild banded mongooses. *Scientific Reports* **6**, 20013. doi:10.1038/srep20013.

Jordan N.R., Cherry M.I., & Manser M.B. (2007) Latrine distribution and patterns of use by wild meerkats: implications for territory and mate defence. *Animal Behaviour* **73**(4), 613-622. doi: 10.1016/j.anbehav.2006.06.010

Kutsukake N., & Clutton-Brock T.H. (2007) The number of subordinates moderates intrasexual competition among males in cooperatively breeding meerkats. *Proceedings of the Royal Society B: Biological Sciences*, **275**(1631), 209-216. doi:10.1098/rspb.2007.1311

Maag N., Cozzi G., Seager D., Manser M., Sickmüller A., Hildebrandt T.B., Clutton-Brock T., & Ozgul A. (2023) Dispersal-induced social stress prolongs gestation in wild meerkats. *Biology Letters*, **19**(6), 20230183. doi:10.1098/rsbl.2023.0183.

Moss, A.M., Clutton-Brock, T.H., & Monfort, S.L. (2001) Longitudinal gonadal steroid excretion in free-living male and female meerkats (*Suricata suricatta*). *General and Comparative Endocrinology*, **122**(2), 158-171. doi:10.1006/gcen.2001.7622

Russell, A. F., Clutton-Brock, T. H., Brotherton, P. N. M., Sharpe, L. L., McIlrath, G. M., Dalerum, F. D., Cameron, E. Z., & Barnard, J. A. (2002). Factors affecting pup growth and survival in co-operatively breeding meerkats *Suricata suricatta*. *Journal of Animal Ecology*, *71*(4), 700–709. https://doi.org/10.1046/j.1365-2656.2002.00636.x

Spong G.F., Hodge S.J., Young A.J., & Clutton-Brock T.H. (2008) Factors affecting the reproductive success of dominant male meerkats. *Molecular Ecology*, **17**(9), 2287-2299. doi:10.1111/j.1365-294X.2008.03734.x

Thorley, J., Duncan, C., Manser, M. B., & Clutton-Brock, T. (2025a) Linking climate variability to demography in cooperatively breeding meerkats. *Ecological Monographs*, **95**(2), e70021. doi:10.1002/ecm.70021

Thorley, J., Duncan, C., Gaynor, D., Manser, M. B., & Clutton-Brock, T. (2025b) Disentangling the effects of temperature and rainfall on the population dynamics of Kalahari meerkats. *Oikos*, **25**(5), e10988. doi:10.1111/oik/10988

Veylit L., Sæther B.-E., Gaillard J.-M., Baubet E., & Gamelon M. (2021) Many lifetime growth trajectories for a single mammal. *Ecology and Evolution*, **11**(21), 14789-14804. doi:10.1002/ece3.8164
